# Emerging phagocytosis checkpoints in cancer immunotherapy

**DOI:** 10.1038/s41392-023-01365-z

**Published:** 2023-03-07

**Authors:** Yu’e Liu, Yanjin Wang, Yanrong Yang, Linjun Weng, Qi Wu, Jin Zhang, Pengcheng Zhao, Lan Fang, Yufeng Shi, Ping Wang

**Affiliations:** 1 Tongji University Cancer Center, Shanghai Tenth People’s Hospital of Tongji University, School of Medicine, Tongji University, Shanghai, 200092 China; 2 Department of Nephrology, Shanghai East Hospital, Tongji University School of Medicine, Shanghai, China; 3 Department of Pharmacology and Toxicology, University of Mississippi Medical Center, 39216 Jackson, MS USA; 4 Clinical Center for Brain and Spinal Cord Research, Tongji University, Shanghai, 200092 China

**Keywords:** Tumour immunology, Cancer microenvironment, Cancer therapy

## Abstract

Cancer immunotherapy, mainly including immune checkpoints-targeted therapy and the adoptive transfer of engineered immune cells, has revolutionized the oncology landscape as it utilizes patients’ own immune systems in combating the cancer cells. Cancer cells escape immune surveillance by hijacking the corresponding inhibitory pathways via overexpressing checkpoint genes. Phagocytosis checkpoints, such as CD47, CD24, MHC-I, PD-L1, STC-1 and GD2, have emerged as essential checkpoints for cancer immunotherapy by functioning as “don’t eat me” signals or interacting with “eat me” signals to suppress immune responses. Phagocytosis checkpoints link innate immunity and adaptive immunity in cancer immunotherapy. Genetic ablation of these phagocytosis checkpoints, as well as blockade of their signaling pathways, robustly augments phagocytosis and reduces tumor size. Among all phagocytosis checkpoints, CD47 is the most thoroughly studied and has emerged as a rising star among targets for cancer treatment. CD47-targeting antibodies and inhibitors have been investigated in various preclinical and clinical trials. However, anemia and thrombocytopenia appear to be formidable challenges since CD47 is ubiquitously expressed on erythrocytes. Here, we review the reported phagocytosis checkpoints by discussing their mechanisms and functions in cancer immunotherapy, highlight clinical progress in targeting these checkpoints and discuss challenges and potential solutions to smooth the way for combination immunotherapeutic strategies that involve both innate and adaptive immune responses.

## Introduction

Generally, cancer cells will be eradicated by the complex system in the human immune system, but they develop resistance to the antitumor immune response to evade the immune surveillance. Cancer immunotherapy has revolutionized the oncology landscape as it utilizes patients’ own immune systems in combating cancer cells. It can be realized in two broad manners: immune checkpoints-targeted therapy and the adoptive transfer of manipulated immune cells. Both manners manipulate the immune system to recognize and attack cancer cells.^1^ Immune checkpoint inhibitors, such as programmed cell death ligand 1 (PD-L1) or cytotoxic T-lymphocyte-associated protein 4 (CTLA-4) antibodies and agonists of costimulatory molecules that override the inhibitory pathways to unleash the immune function, have achieved success in various clinical trials but still face problems such as low response rates, high costs, and nonspecific toxicity.^2–4^ Adoptive transfer of cells basically includes genetically engineered cells including chimeric antigen receptor (CAR)-T cells and many other cells, e.g., multipotent mesenchymal stem cells engineered to express a cytokine and characteristics of other manipulated cells.^5,6^ In a word, cancer immunotherapy has experienced remarkable advances since the clinical success of immune checkpoint blockade and CAR-T-cell therapies in recent years. It has become an innovative treatment and a powerful clinical strategy due to its incomparable advantages over traditional antitumor therapy including surgery, radiotherapy, and chemotherapy.

**Fig. 1:**
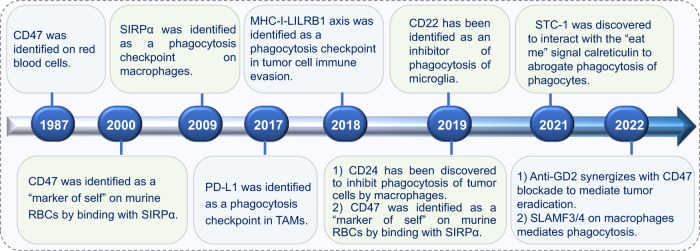
Discovery of phagocytosis checkpoints

**Fig. 2:**
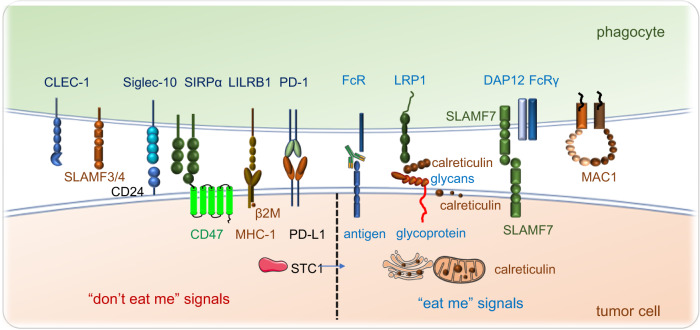
Phagocytosis checkpoints in cancer immunotherapy. Phagocytosis of tumor cells by macrophages is regulated by many “eat me” (pro-phagocytosis) and “don’t eat me” (anti-phagocytosis) signals. The expression of “don’t eat me” signals on tumor cells including CD47, CD24, PD-L1, MHC-I and STC-1 protect tumor cells from phagocytic clearance by interacting with their receptors on phagocytes. The working pathways are CD47-SIRPα, CD24-Siglec-10, MHC-1(B2M)-LILRB1, and PD-L1-PD-1. The high expression of tumor STC-1 traps the calreticulin in mitochondria and ER thus reducing the quantity of calreticulin on the cell surface, impairing phagocytosis and antigen processing and presentation, also leading to weak T cell response. Other anti-phagocytosis receptors such as SLAMF3, SLAMF4, FcγRIIB, and CLEC-1 facilitate the phagocytosis of tumor cells by phagocytes. The “eat me” signals such as calreticulin bind with the membrane glycans and are located on the cancer cell surface. It interacts with the lipoprotein receptor-related protein 1 (LRP1) receptor present on phagocytes. It seems that SLAMF7 expressed on tumor cells and MAC-1 on macrophages are both critical for inducing tumor phagocytosis, while the clear mechanism of SLAMF7-induced phagocytosis is under investigation

Most previously developed immunotherapies worked primarily by stimulating adaptive immunity, especially by revitalizing and boosting T cell responses. However, emerging studies have manifested that innate immune checkpoints expressed on the antigen-presenting cells (APCs) play a critical role in the immune evasion. These checkpoints detect and eliminate cancer cells by phagocytosis and inhibit the innate immune response. Innate immune cells that function as APCs, including macrophages, monocytes, dendritic cells (DCs), and natural killer (NK) cells are the first line of immune defense system. They establish proinflammatory responses to foreign invaders and repair damaged tissues. Cancer cells evade clearance by macrophages via overexpressing the anti-phagocytic membrane proteins termed “don’t eat me” signals, including cluster of differentiation 47 (CD47),^7^ cluster of differentiation 24 (CD24),^8^ PD-L1,^9^ the beta-2 microglobulin (β2M) subunit of the major histocompatibility class I complex (MHC-I),^10^ stanniocalcin 1 (STC-1),^11^ and GD2^12^ (Figs. 1, 2). Phagocytosis is often facilitated by intrinsic “eat me” signals that function as ligands for phagocytic receptors, which can trigger extensive remodeling of the cytoskeleton and engulf the target.

In this review, we summarize the phagocytosis checkpoints discovered to date, including basic knowledge, relevant pathways, and functions in cancers and the tumor microenvironment. We also discuss the expression and functions of these checkpoints in innate and adaptive immune responses. Finally, we highlight clinical progress in targeting these phagocytosis checkpoints, listing challenges and potential solutions for targeted cancer immunotherapy. We hope that this comprehensive review will not only help understand the current status of phagocytosis checkpoint research but also aid in the development of combinatorial treatment approaches, such as immunotherapy, that build on chemotherapy by targeting phagocytosis checkpoints.

## Basic knowledge of phagocytosis checkpoints

### CD47

The CD47-signal regulatory protein alpha (SIRPα) axis, identified in the late 2000s, is the first tumor phagocytosis checkpoint and is a typical myeloid-specific immune checkpoint that works directly via innate immunity.^13^ CD47, which serves as a “don’t eat me” signal on tumor cells, inhibits phagocytosis by macrophages in the immune system.^14–16^ Myriad CD47 inhibitors and antibodies are under investigation, and many of them are in clinical trials.^17,18^ In clinical trials, striking responses have been achieved for some solid tumors and hematologic malignancies upon CD47 inhibition.^19,20^ Moreover, CD47-SIRPα signaling relies on the phagocytic function of macrophages, which are the most abundant infiltrating leukocytes in tumors. Therefore, targeting CD47 likely represents a turning point in cancer immunotherapy. An elaborate discussion of CD47 regulation and its function in cancer immunotherapy will be presented in the following sections.

#### A brief history of CD47

CD47 was originally identified in 1987 on red blood cells (RBCs).^21^ Until 2000, CD47 was viewed as a “marker of self” on murine RBCs that binds to SIRPα on phagocytes.^22,23^ It was confirmed as a tumor phagocytosis checkpoint that delivers a “don’t eat me” signal during immune evasion in 2019, and CD47-targeting antibodies and inhibitors are currently in clinical trials.

#### Structure of CD47

In the immune system, CD47 is the only 5-transmembrane (5-TM) receptor.^24^ It contains three parts: a heavily glycosylated N-terminal extracellular domain (ECD), a 5-TM spanning domain and a short C-terminal domain (CTD).^25^ The ECD includes a V-set immunoglobulin superfamily domain binding to SIRPα. The CTD is alternatively spiced into 4 isoforms named from type I to type IV, which vary in expression in different cells.^26^ All the above structures and splicing isoforms are crucial for CD47 function.^24^

#### CD47 binding proteins

CD47 interacts with other extracellular proteins located on the membrane and inside cells. Most of its functions are attributed to its binding partners. The best-studied binding proteins of CD47 are thrombospondin 1 (TSP-1) and SIRPα. In addition to SIRPα, SIRPγ also binds to CD47 due to the similarity of its extracellular domain to SIRPα, but it has a tenfold lower affinity.^27,28^

TSP-1 was the first identified ligand for CD47.^29,30^ It interacts with CD47 via the RFYVVMWK sequence (4N1K) in the C-terminal of the CBD domain.^31^ The CD47-TSP-1 interaction inactivates the vascular endothelial growth factor receptor 2 (VEGFR2) and suppresses angiogenesis to inhibit tumor growth; thus, TSP-1 has also been viewed as a potent inhibitor of tumor growth and metastasis.^32^ The CD47-TSP-1 interaction also inhibits inflammatory responses such as cytokine secretion.^33,34^ TSP-1 deficiency in macrophages limits their phagocytic capacity.^35^ Furthermore, the interaction of CD47 and TSP-1 enhances the regeneration of stem cells by upregulating transcription factors of stem cells such as KLF4, Sox2, c-Myc and Oct4.^36^

SIRPα was identified as an endogenous ligand of CD47.^37^ It is also a transmembrane glycoprotein that is mainly expressed on macrophages, monocytes, and DCs. It contains one transmembrane domain, three lg-like domains, and four tyrosine phosphorylation sites. In the cytoplasmic tail, there are two immune receptor tyrosine-based inhibitor motifs (ITIMs).^38^ The interaction of SIRPα and CD47 is mediated by the N-terminal of SIRPα and the single lg-V domain of CD47.

#### The regulation of CD47

CD47 expression is regulated at different levels. First, transcription factors such as Myc,^39^ hypoxia-inducible factor-1 (HIF-1),^40^ and nuclear respiratory factor 1 (NRF-1)^41^ potentiate nuclear factor kappa B (NF-κB) CD47 expression.^42^ Moreover, cytokines, including tumor necrosis factor alpha (TNF-α),^43,44^ interferon-γ (IFN-γ)^45^ and interleukin,^46,47^ enhance CD47 expression. Conversely, various microRNAs and long noncoding RNAs (lncRNAs) negatively regulate CD47.^48^ At the posttranslational level, the pyroglutamylation and glycosylation of CD47 have been studied thoroughly.^49^ Lautenberg et al., Shana et al., and our group reported that CD47 is a substrate of QPCTL and that the N-terminal region of CD47 is pyroglutamylated. The pyroglutamylation of CD47 is catalyzed by QPCTL;^50–52^ this modification is critical for the recognition of CD47 by SIRPα and contributes to their interaction.^49,51^

#### Cellular function of CD47

CD47 plays a critical role in various biological and pathological processes. It either promotes or suppresses cell proliferation depending on cell status and type, and the expression of CD47 promotes cell proliferation in glioma cells but not in normal astrocytes.^53^ Moreover, CD47 enhances aerobic glycolysis, and CD47 activation contributes to the PI3K/Akt/mTOR oncogenic pathway.^54^

### PD-L1

PD-L1, a ligand of programmed cell death protein 1 (PD-1), is a well-recognized immune checkpoint expressed on tumor cells. Antibodies targeting PD-1/PD-L1 have been widely used clinically for various types of tumors, and PD-1-PD-L1 blockade ushered in a new era of tumor treatment. Hence, it is a breakthrough of targeting the PD-1-PD-L1 pathway in tumor treatment.

In 1999, Chen’s team discovered a B7 homologous transmembrane protein, B7-H1 (now known as PD-L1).^55^ Later, it was found that PD-L1 is a ligand of PD-1, which clarified the negative immune regulation function of PD-L1 and highlighted its potential for application in tumor treatment.^56^ In 2002, PD-L1 was demonstrated to promote T cell apoptosis, and a B7-H1 antibody was applied to inhibit tumor growth, which demonstrated that PD-L1 functions in tumor immune escape for the first time.^57,58^ Since then, the effectiveness of PD-L1 antibody therapy has been witnessed by successive clinical trials.

*Cd274* gene encodes PD-L1 protein, it is located on chromosome 9 of human and 19 of mouse. It is composed of a transmembrane region, typical immunoglobulin V-like plus C-like extracellular domains, and a short tail in cytosol.^59^ PD-L1 presents on a variety of hematopoietic cells, including DCs, macrophages, B cells and T cells, and other nonhematopoietic cells, such as vascular endothelial cells. Besides that, PD-L1 is also usually overexpressed in various types of cancer cells.^60^ PD-L1 expression on normal cells facilitates the regulation of immune responses in the periphery, but its overexpression on cancer cells protects cancer cells from immune surveillance.

PD-L1 expression is regulated by multiple factors at the genomic, transcriptional and posttranslational levels. For example, inflammatory signaling regulates PD-L1 expression. PD-L1 expression can be induced by both type I and type II interferons, TNF-α, and vascular endothelial growth factor (VEGF).^60^ Type I interferons, IFN-α and IFN-β stimulate PD-L1 expression.^61^ In prostate cancer and kidney cancer, TNF-α upregulates PD-L1 expression by activating NF-κB pathway.^62,63^ Type II interferon IFN-γ binds to IFNGR and triggers JAK-STAT1-IRF1 to modulate PD-L1 expression. Moreover, IL-6 activates the JAK-STAT3 or MEK/ERK signaling pathway to upregulate PD-L1 expression,^64^ and TGF-β also regulates the expression of PD-L1 in cancer cells.^64^ In addition to inflammatory factors, oncogenic pathways such as the epidermal growth factor receptor (EGFR), HIF-1, Myc, NF-κB, PTEN/PI3K-AKT, and mitogen-activated protein kinase (MAPK) pathways play vital roles in PD-L1 expression.^65^ The mechanisms by which PD-L1 expression is regulated were reviewed in another excellent review,^66^ and this article mainly focuses on phagocytosis and the PD-1-PD-L1 axis.

### MHC-I

Major histocompatibility complex (MHC) is a cluster of closely linked genes that are highly polymorphic and located in a specific region of the mammalian chromosome. The molecules encoded by these genes are expressed on the surface of all nucleated cells and platelets but not on RBCs.^67^ They are involved in antigen presentation, governing intercellular recognition and the induction of immune responses. The basic function of MHC is to distinguish “self” and “non-self” and present the tumor-associated antigens (TAAs) to T cells to activate the adaptive immune response.^68^ Moreover, MHC-I on the surface of tumor cells binds to leukocyte immunoglobulin-like receptor subfamily B 1 (LILRB1) on the surface of macrophages to promote tumor cell escape from macrophage phagocytosis. Therefore, MHC-I-LILRB1 is another phagocytosis checkpoint in cancer immunotherapy.

#### A brief history of MHC-I

MHC genes were discovered in 1937. The key to successful transplantation is histocompatibility between the host and the donor, and the genes that mediate this recognition are called compatibility genes. They are closely linked on the same chromosome, and their product is the MHC, also known as the transplant antigen, which is the main determinant of transplant rejection.^69^ The MHC locus encodes classical MHC-I, MHC class II (MHC-II) and nonclassical MHC-I molecules. MHC-I, as the first human leukocyte antigen product, controls the immune response induced by protein antigens.^70^

#### Structure of MHC-I

In humans, MHC refers to human leukocyte antigen (HLA), which includes classical HLA-I, HLA-II, and nonclassical HLA-III molecules. HLA-I binds to and presents endogenous antigens. HLA is the most complex and polymorphically rich genetic system in humans by far,^71^ and it possesses a tremendous number of alleles to achieve the most appropriate immune response to pathogens and enable adaptation to a variable internal and external environment. Classical MHC-I is a heterodimer composed of an α heavy chain and a β2m light chain. The former chain contains three sites: three extracellular structural regions (α1, α2, and α3),^72^ a membrane-penetrating region and a cytoplasmic region. The α3 structural region is structurally homologous to the constant region of Ig and is the site of binding to CD8 on the surface of T cells.^73^ The α1 and α2 structural regions interact to form the antigen binding site of MHC-I. The binding groove is closed at both ends. The middle part of the antigenic peptide is generally elevated and recognized by the T-cell receptor (TCR) as a T-cell epitope. β2m is a soluble protein that cannot pass through the cell membrane. The sequence of amino acid for β2m is highly conserved, with minimal differences among species, and can be substituted for each other. The main function of β2m is to stabilize MHC-I molecules and facilitate their cell-surface expression.

#### MHC-I binds to LILR1 and LILRB2 on phagocytes to inhibit phagocytosis

MHC-I on tumor cells binds to LILRB1 and LILRB2, which are members of the LILR family,^74^ which belongs to the inhibitory class of the LIR receptor subfamily. LILRBs are overexpressed typically in immunosuppression-related cells, such as tolerogenic DCs and the immunosuppressive M2-type macrophages.^75^ LILRB1 expression is significantly increased after the differentiation of human monocytes into immature DCs. Subsequently, MHC-I molecules are upregulated for antigen-presenting functions, whereas LILRB1 is downregulated.^76^ Recently, it was found that the binding between β2m of MHC-I expressed on the surface of tumor cells and LILRB1 on the surface of tumor-associated macrophages (TAMs) inhibits the phagocytic activity of TAMs, leading to decreased immune surveillance and enhanced immune escape of tumor cells.^10^

#### Regulation of MHC-I

Dozens of genes have been reported to positively or negatively regulate MHC-I expression.^77^ The positive regulators include interferon signaling,^78^ mRNA processing and splicing,^79^ endoplasmic reticulum (ER) quality control,^80^ etc. The negative regulators include mammalian target of rapamycin (mTOR) regulation, mRNA capping and translation,^81^ polycomb repressive complex 2 (PRC2), the ubiquitin system,^82^ and a myriad of endo-lysosomal trafficking factors that are likely critical for internalizing MHC-I and its lysosomal degradation.^83^ MHC-I is removed from the cell surface when an HIV-1-encoded protein Nef is present.^84^ MIIP, CAMSAP3, SLC6A3 and KCTD19 were found to significantly inhibit Nef-induced MHC-I downregulation.^85^ Moreover, the 3’UTR of HLA-A2 mRNA has been found to bind the ubiquitin E3 ligase MEX-3C, which leads to its RING-dependent degradation.^86^

### CD24

The CD24-sialic acid-binding immunoglobulin-like lectin-10 (Siglec-10) axis is known to protect the body from a lethal response involving pathological cell death.^87^ Recent studies indicated that blocking the binding of CD24 and Siglec-10 with a CD24 antibody significantly enhances the recognition of CD24-expressing tumor cells by macrophages, and after CD24 antibody treatment, the growth of murine orthotopic tumors was inhibited strikingly. Therefore, CD24 has been widely studied and explored as a new antitumor phagocytosis checkpoint.

#### A brief history of CD24

As a heat-stable antigen, CD24 was first found in 1978, and it was thought to be expressed on the membrane of immature B cells, T lymphocytes, and activated granulocytes as a marker of the differentiation and maturation of immune cells.^88^ In 2019, CD24, serving as a “don’t eat me” signal on tumor cells, was found to inhibit the phagocytosis of macrophages in the innate immune system.^8^ CD24 mediates adhesion between cells, cells and substrates and also functions in cell recognition, activation, signal transduction, proliferation, differentiation, extension and movement.^89^ Recently, increasing evidence has proven that the expression of CD24 on the surface of tumor cells, in contrast to that in adjacent tissue, is significantly elevated, which is positively associated with the occurrence and development of tumors.

#### Structure of CD24

The *CD24* gene encodes a glycosylated protein and is located on chromosome 6q21. As a single-chain sialoglycoprotein, mature CD24 is a short peptide with only 30 amino acids. There are one or more O-linked glycosylation sites in the mature peptide backbone and four potential N-linked glycosylation sites in CD24.^90^ Thus, the glycosylation modifications of CD24 vary substantially among different cell types, resulting in molecular masses from 35 kDa to 45 kDa. Highly glycosylated CD24 requires anchoring on lipid rafts within the plasma membrane through a glycosyl-phosphatidyl-inositol (GPI) anchor protein.^91^

#### CD24 binding proteins and corresponding functions

Primarily, CD24 as a GPI-anchored protein, is located in the cell membrane in both normal and cancer cells, but is also distributed in the cytoplasm and nucleus in some cancer cells. The functions of CD24 on the membrane depend on its binding proteins. It binds to different proteins, such as Siglec10, Siglec E, platelet (P)-selectin, and L1-cell adhesion molecule (L1-CAM), to perform a variety of functions. Since only CD24-Signlec 10 is related to phagocytosis function, we focus on this binding protein in the following part.

CD24 binds to Siglec 10 on macrophages to avoid phagocytosis. Siglec10 is an immunosuppressive receptor, and the interaction between CD24 and Siglec10 significantly reduces the damage associated with damage-associated molecular pattern (DAMP)-related inflammatory responses, including liver injury^87^ and sepsis; this interaction also reduces antigen sensing at the cell surface or in the endosomal compartment and reduces the phagocytosis of tumor cells by tumor-associated macrophages, thus promoting tumor progression.^92,93^ Moreover, this interaction participates in the establishment of maternal immune tolerance in early pregnancy^94^ and is also involved in autoimmune diseases^95^ and graft-versus-host disease.^96^

#### The regulation of CD24

The expression of CD24 in tumors is regulated by a variety of factors. CD24 is upregulated by HIF1α in human bladder cancer,^97^ androgen receptor in urothelial carcinoma,^98^ DNA methyltransferase,^99^ estrogen receptor^100^ and truncated glioma-associated oncogene homolog 1^101^ in breast cancer. CD24 expression is negatively regulated by Twist in breast cancer,^102^ β-catenin/TCF in colorectal cancer,^103^ miR34a^104^ and miR-146a^105^ in oral squamous cell carcinoma, and histone deacetylase (HDAC)^99^ in breast cancer. As a highly glycosylated GPI-anchored protein, the localization of CD24 on the membrane is regulated by the proteins related to both the synthesis of N and O sugars and GPI assembly, such as PIGN, PIGP, and PGAP2.

#### Intracellular function of CD24

CD24 can be accumulated in the cytoplasm due to defects in the GPI system, such as loss of function of GPI assembly proteins, weak GPI anchor attachment, errors in the synthesis of CD24 in the ER, and the inclusion of CD24 in microvesicles.^106^ Localization of CD24 in cytosol also affects tumor cell development.^107^ CD24 in the cytoplasm of tumor cells inactivates and destabilizes p53 by disrupting the ARF-NPM interaction, which protects mutant p53 from degradation.^108^ The CD24-p53 axis also suppresses the tumorigenesis by maintaining intrahepatic macrophages, which can remove hepatocytes with DNA damage in hepatocellular carcinoma (HCC).^107^

The functions of cytoplasmic CD24 in tumor proliferation and metastasis are controversial. Mierke et al. reported in 2004 that CD24 enhances cell invasion through different pathways, such as increasing contractility and stimulating cell adhesion to fibronectin and collagen I and IV.^109^ However, a later study showed that intracellular CD24 suppresses tumor cell invasion and metastasis by influencing the posttranscriptional regulation of BART via G3BP RNase activity.^110^

### STC-1

STC-1 was identified a phagocytosis checkpoint in 2021.^11^ STC-1 was first discovered in the corpuscles of the stannius of bony fishes,^111^ and its homologous genes in mammals, STC-1 and STC-2, were subsequently cloned.^112^ STC-1 is widely expressed in the ovary, prostate, bladder, kidney, adrenal gland, lung, heart, uterus, and pituitary gland in mammals,^113^ and its expression is upregulated in breast cancer, which potentiates invasiveness of breast cancer via JNK-/c-Jun pathway.^114^

STC-1, as a glycoprotein, functions in the regulation of serum calcium and phosphate homeostasis.^112^ It plays a more complex role in pregnancy, lactation, angiogenesis, organogenesis, proliferation, apoptosis, ischemia, and tumorigenesis.^115,116^ STC-1 acts as a SUMO E3 ligase in the SUMOylation cycle, and interacts with proteins located in the nucleus, endoplasmic reticulum, mitochondria, cytoplasm, membrane and secreted proteins.^117^ In diabetic nephropathy, STC-1 inhibits BNIP3 via AMPK/SIRT3 pathway and thus ameliorates renal injury.^118^ STC-1 also functions in the oxygen-induced retinopathy (OIR) stress response and development of pathologic vascular features in rodent OIR models by regulating VEGF levels.^119^ Emerging evidence has shown that STC-1 is present in various human cancer cells. It is closely associated with the efficacy of immunotherapy and is further related with patient survival negatively in various cancer types.^11^

### GD2

Besides proteins, carbohydrates and lipids are also involved in the regulation of phagocytosis. GD2, a disialoganglioside, was identified as a tumor antigen of neuroblastoma in the 1980s; it is consistently overexpressed in neuroblastoma, sarcomas, gliomas, and neuroendocrine tumors and is regarded as the most promising tumor antigen.^120^ Anti-GD2 antibody has prolonged the survival of patients suffering from neuroblastoma.^121,122^ The role of GD2 as a cancer target has been reviewed elsewhere.^123^

GD2 is composed of five monosaccharides and contains glucose, galactose and two sialic acid residues linked to ceramide. GD2 is embedded in the outer plasma membrane via its ceramide tail, and the carbohydrate moiety is exposed to the extracellular space.^124^ GD2 expression is low in normal tissues and restricted to the brain, spinal cord, and skin melanocytes.^125^ The role of GD2 in normal development is thought to be involved in neural differentiation and repair,^126^ but clear mechanisms deserve further investigation.

As a complex ganglioside, GD2 regulates cell-cell recognition and signal transduction via specific binding lectins like Siglecs.^127^ GM2/GD2 synthase (B4GALNT1) deficient mice exhibit decreased central myelination, demyelination in peripheral nerves, and axonal degeneration in the nervous system, indicating the complex gangliosides role in the maintenance of the integrity of axons and myelin.^128^ Moreover, mice with GM2/GD2 synthase deficiency developed progressive behavioral neuropathies, indicating GM2/GD2 maintains the normal neural physiology.^129^ The function of GD2 in normal cellular physiology is not clearly illustrated, but GD2 augments cancer cell proliferation, adhesion, migration and invasion, and confers resistance to apoptosis.^123^

### “Don’t eat me” receptors

In addition to the above “don’t eat me” signaling molecules that are highly expressed on cancer cells, there are many other “don’t eat me” receptors expressed on immune cells, including but not limited to SIRPα, Siglec-10, and LILRB1, which were mentioned in the previously described signaling pathways.

#### CD22

**Fig. 3:**
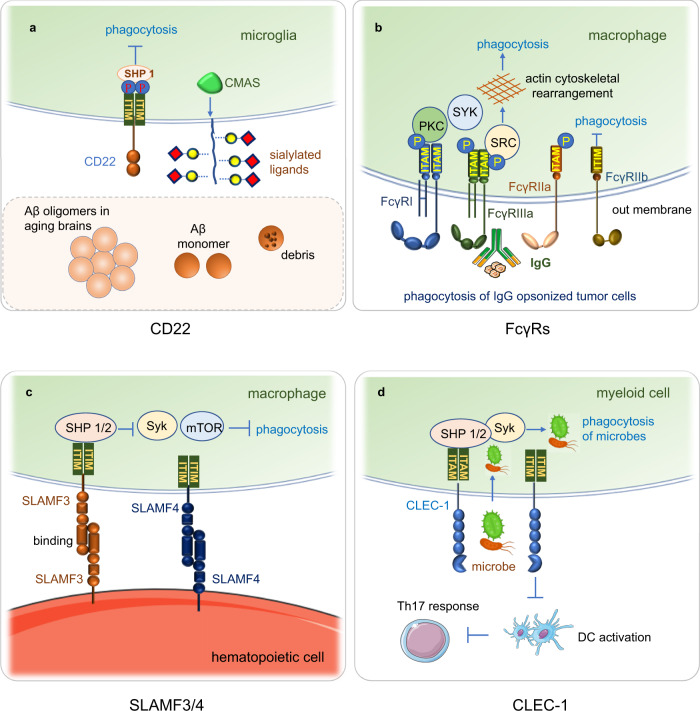
The phagocytosis receptors CD22, Fc receptors, SLAMF3/4 and CLEC1. **a** CD22 binds α2,6-linked sialic acid and recruits tyrosine phosphatase SHP-1 to inhibit the phagocytic capacity of microglia. The anti-CD22 treatment enhanced clearance of injected oligomeric amyloid-β (Aβ), myelin debris and α-synuclein fibrils in aging brains. CMAS is a key synthase functioning in sialic acid synthesis, related to CD22 function. **b** FcγRIIb, FcγRI, FcγRIIIa, and FcγRIIa are expressed on macrophages. FcγRs crosslink IgG immune complex triggers phosphorylation of their ITAMs and activates kinases of SYK, SRC and PKC pathway, kinase activation leads to actin remodeling, which is crucial for phagocytosis of the IgG immune complex. FcγRIIB is the only phagocytosis-inhibitory receptor, and the other family members are phagocytosis-activating receptors within the human FcγR family. FcγRIIB contains an ITIM in its cytoplasmic region, and the phosphorylation and activation of the ITIM recruit the phosphatases SHP1 and SHP2 and inhibit downstream phagocytosis. **c** SLFRs are ubiquitously expressed in hematopoietic cells. SLAMF3 and SLAMF4 were identified as “don’t eat me” receptors on macrophages. They inhibit “eat me” signals, such as lipoprotein receptor-related protein 1 (LRP1) -mediated activation of mTOR and Syk to macrophages through SH2 domain-containing phosphatases and hematopoietic cells without SFRs are easily phagocyted by macrophages. **d** CLEC-1 is expressed primarily by myeloid cells, CLEC-1 on human DC dampens DC activation and restrains downstream Th17 responses, CLEC-1 is a novel myeloid immune checkpoint limiting tumor cells’ phagocytosis and tumor antigen presentation. CLRs binding to microbial surfaces influence phagocytosis by promoting inflammatory signals and triggering intracellular signaling to induce phagocytosis of microbes

CD22 is expressed exclusively on B cells and is a cell surface sialoglycoprotein, it regulates the proliferation and function of B cells, acting as an inhibitory coreceptor of the B-cell antigen receptor (BCR).^130^ CD22 is present in the cytoplasm of progenitor and pre-B cells in early B-cell development and translocates to the surface of B cells as they mature.^131^ CD22 expression is highest in mature B cells. Therefore, it is an appealing therapeutic target for B-cell malignancies and autoimmune disorders. CD22 has been identified as an inhibitor of phagocytosis in microglia (Fig. 3a).^132^

The expression and function of CD22 are regulated by many molecules. Its synthase CMAS, a key enzyme in sialic acid synthesis, and PTPN6, which encodes SHP-1, are related to CD22 function,^132^ and spleen tyrosine kinase (Syk), phospholipase Cγ2 (PLCγ2), phosphoinositide 3-kinase (PI3K), Grb2, and Shc are the binding proteins of the CD22 cytoplasmic tail in response to BCR signaling.^133^

Ligands of CD22 have been identified on B cells, microglia,^132^ DCs and T cells.^134^ CD22 on B cells binds to α2,6-linked sialic acid on microglia and recruits the tyrosine phosphatase SHP-1 to inhibit the phagocytic capacity of microglia.^132^ Anti-CD22 treatment enhanced the clearance of injected oligomeric amyloid-β (Aβ), myelin debris and α-synuclein fibrils in the aging brain. Long-term CD22 blockade changes the transcriptional profile of microglia, including genes associated with microglial homeostasis, and improves cognitive function in aged mice.^132^ CD22-mediated phagocytosis in TAMs and in cancer immunotherapy requires further study. Moreover, DCs and bone marrow-derived immature DCs (iBMDCs) express glycan ligands of CD22, and iBMDCs induce strong inhibition of BCR-induced B-cell proliferation via a CD22-dependent mechanism.^135^ iBMDCs also suppress the proliferation and differentiation of B-cell subsets during Toll-like receptor (TLR) stimulation.^136^ Therefore, CD22 is a regulator of receptors that mediate both adaptive and innate immune responses. CD22 binds to ligands on T cells and affects T-cell activation. In addition, CD22 regulates B-cell responses to T-cell-independent type 2 antigens (TI-2 Ags). CD22 also negatively modulates TLR pathway, and CD22^−/−^ B cells showed enhanced proliferative ability in response to TLR3, TLR7, and TLR9 agonists.^137,138^ Mechanistically, CD22 inhibits TLR signaling via intracellular signaling in B cells because the natural ligands for CD22 do not appear to affect proliferative responses to TLR agonists.^138^

CD22 plays a critical role in maintaining B-cell homeostasis in human immunity.^139^ The phosphorylated ITIMs of CD22 recruit the tyrosine phosphatase SHP-1 during antigen-mediated BCR crosslinking.^140,141^ CD22 knockout B cells induce responses, such as the intracellular calcium mobilization required for the proliferation and antibody production of B cells.^142–144^ CD22 also regulates the migration of recirculating B cells to the bone marrow,^145^ and CD22-deficient B cells inhibit homing to Peyer’s patches by reducing integrin expression via the CD22-Shp1 axis.^146^

CD22 is one of the most common antigens and is highly expressed in hematological malignancies, including human B-cell lymphomas and leukemias.^147–149^ Exon 12 depletion in infant B-precursor leukemia cells promotes their growth and survival.^150^ Moreover, CD22 conduces to protecting against pathogenic infection, and CD22 deficient mice are extremely sensitive to infection.^151^ In addition, CD22 expression is closely related to autoimmune disease, and CD22 levels are decreased in patients with systemic lupus erythematosus (SLE) and increased after effective treatment.^152,153^

#### Fc receptors

Fc receptors (FcRs) are cell-surface receptors present on several hematopoietic cells that specifically recognize the Fc region of immunoglobulin (Ig) to regulate phagocytosis and antibody-dependent cell-mediated cytotoxicity (ADCC).^154^ Generally, type I Fc common gamma receptors (FcγRs) are divided into activating or inhibitory subtypes. The activating FcγRs include FcγRI, FcγRIIa, FcγRIIc and FcγRIIIa, all of which contain immunoreceptor tyrosine activating motifs (ITAMs); FcγRIIB is the only phagocytosis-inhibitory receptor, and the others are phagocytosis-activating receptors within the human FcγR family.^155^ FcγRIIB comprises an ITIM in its cytoplasmic region,^156^ and the phosphorylation and activation of the ITIM recruit the phosphatases SHP1 and SHP2 and inhibit phagocytosis in their downstream (Fig.3b).

FcRs are present on different immune cells, such as monocytes, macrophages, DCs, and neutrophils, and the unique expression patterns of individuals or combinations of FcγRs balance cellular immune responses.^156^ FcγRIIb, FcγRI, FcγRIIIa, and FcγRIIa are expressed on macrophages. IgG immune complexes activate FcγR signaling for different subtypes of IgG, with complex binding specificity and affinity.^157^ After ligation of these immune complexes, ITAMs are phosphorylated by kinases of the SRC family, which recruits SYK-family kinases, followed by the activation of many downstream targets to activate the immune response, ADCC or phagocytosis.^158^ FcR function is important for the treatment of cancers especially when using the immune checkpoint-blocking drugs in cancer therapy.^159–161^ It may be possible to selectively exploit FcR activation or immune regulation function by engineering antibodies for different therapeutic environments.

#### Signaling lymphocytic activation molecule (SLAM) family receptors (SFRs)

Signaling lymphocyte activation molecules (SLAMs) are important immune regulatory receptors that have critical functions in immunity, cell survival, lymphocyte development, and cell adhesion.^162^ SLAM family receptors (SFRs) belong to an immunoglobulin superfamily that is expressed ubiquitously on hematopoietic cells, including macrophages, and modulate the activation and cytotoxicity of these cells. They recognize themselves as self-ligands and thus undergo homotypic interactions to constrain macrophage phagocytosis.^163^ Hematopoietic cells without SFRs are easily phagocytized by macrophages. The SFR members LAMF3 and SLAMF4 were identified as “don’t eat me” receptors on macrophages. They inhibit “eat me” signals in macrophages by SH2 domain-containing phosphatases (Fig. 3c). SFRs are markers that distinguish HSCs and their progenitors and prevent the inappropriate phagocytosis of self-HSCs. Mature RBCs express high levels of CD47 to avoid macrophage engulfment. SFRs can work in combination with the CD47 pathway but function independently of CD47 to mitigate macrophage phagocytosis.^163^ SLAMF3 is also expressed in cancer cells,^164^ but its function in phagocytosis in cancer immunotherapy remains unclear.

#### C-type lectin-like receptor-1 (CLEC-1)

C-type lectin-like receptors (CLRs) are a family of transmembrane receptors present on myeloid cells primarily. They recognize pathogen moieties for host defense and modify self-antigens. CLRs have at least one C-type lectin-like domain (CTLD) on the cell surface and either a transmembrane domain or a short intracellular signaling tail that boosts interaction with FcRγ that mediates signaling. CLRs binding to microbial surfaces influence phagocytosis by promoting inflammatory signals and triggering intracellular signaling to induce phagocytosis of microbes.^165^ C-type lectin-like receptor-1 (CLEC-1) is a prototypical CLR and an inhibitory receptor present on neutrophils, DCs and myeloid macrophages. CLEC-1 on human DCs dampens DC activation and restrains downstream Th17 responses.^166^ CLEC-1-deficient mice eradicate colorectal tumors by combining with cytotoxic and immunogenic chemotherapy, and CLEC-1 blocking antibodies augment the phagocytosis of CLEC-1 L-positive tumor cells by DCs and macrophages.^167^ CLEC-1 probably signifies a new therapeutic agent to regulate the immune response in transplantation, autoimmunity, and cancer. CLEC-1 is a novel myeloid immune checkpoint that limits tumor cell phagocytosis and tumor antigen presentation (Fig. 3d).^167,168^

## “Eat me” signals

“Eat me” signals are molecules expressed on or released from cells to induce phagocytosis by a phagocyte. Most “eat me” signals are located on the cell surface, but some may be released extracellularly and bind back to the target cell. The lipid phosphatidylserine, the intracellular adhesion molecule ICAM-3, annexin I, calreticulin, cell surface-bound thrombospondin, complement factors, oxidized low-density lipoprotein, and other glycosylation alterations on apoptotic cells are “eat-me signals”.^169^ These signals have been reviewed previously.^170^

The phagocytosis process of tumor cells by macrophages or DCs is modulated by a large number of pro-phagocytosis (“eat me”) and anti-phagocytosis (“don’t eat me”) signals via the receptor-ligand axis. All the abovementioned checkpoints are antiphagocytosis proteins or signaling molecules. The “eat me” signals mainly include tumor-associated antigens generated in response to oncogenic stresses, the ER chaperone protein calreticulin and the glycoprotein SLAMF7.

### Calreticulin

Calreticulin is an ER-resident protein and functions in various cellular processes, such as stress, and it functions as a chaperone and Ca^2+^ buffer to aid in appropriate protein folding and glycosylation.^171^ Calreticulin contributes highly to phagocytosis, the loss of wild-type calreticulin functions favors oncogenesis due to impaired cellular homeostasis in healthy cells and compromised natural and therapy-driven immunosurveillance.

**Fig. 4:**
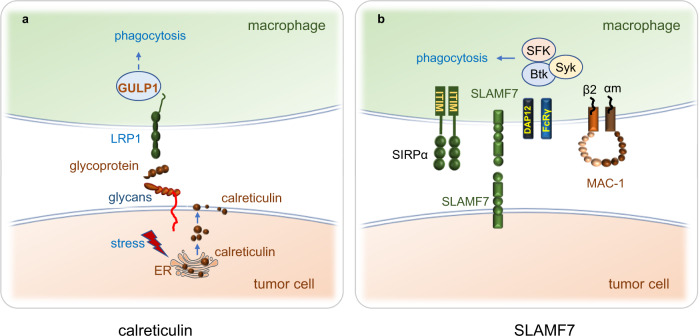
The “eat me” signals calreticulin and SLAMF7. **a** Stressed and dying tumor cells expose calreticulin on the surface of the cell from ER, and cell surface calreticulin binds to LRP1 on the phagocyte. LRP1 may recruit GULP1, an adapter protein LRP1 for regulating further phagocytic processes. **b** SLAMF7 on macrophage binds to MAC-1 on the macrophage, and MAC-1 interacts with FCRγ and DAP12 recruiting Src family Syk, and Btk kinases and promoting phagocytosis. SLAMF7 on macrophages combining with SIRPα on macrophage may affect the CD47-SIRPα axis, SLAMF7 in hematological cancers binds SLAMF7 on phagocytes and is necessary for phagocytosis

Through binding with membrane glycans, calreticulin is anchored to the cancer cell surface, and it interacts with the low-density lipoprotein receptor-related protein 1 (LRP1) receptor present on phagocytes. LRP1 may recruit the adapter protein PTB domain-containing engulfment adapter protein 1 (GULP1) to regulate further phagocytic processes (Fig. 4a). Calreticulin translocates to the cell membrane and serves as an “eat me” signal to promote efferocytosis of apoptotic cells, including damaged, aged, and malignant cells, and leads to the elimination of these cells.^172^ Calreticulin has been demonstrated to be the dominant pro-phagocytic signal in a myriad of human cancers and is counterbalanced by CD47.

### SLAMF7 synergizes with MAC-1 and promotes phagocytosis

SLAMF7, also known as CD319, CS1 or CRACC, is a member of the SLAM family of receptors that are present on both tumor cells^173–175^ and immune cells, including NK cells, B cells, DCs, and activated CD4 and CD8 T cells.^162^ SLAMF7 on macrophages recognizes homotypic SLAMF7 on hematopoietic cells to mediate phagocytosis. SLAMF7-deficient macrophages, but not macrophages deficient in other SFRs, have a defect in phagocytosis. SLAMF7 on macrophages interacts with integrin macrophage-1 antigen (MAC-1) on macrophages to promote the phagocytosis of cancer cells by macrophages. MAC-1 is a complement receptor (CR3) containing α-subunit CD11b (αm) and β-subunit CD18 (β2); it interacts with ITAM,^175^ FcRγ and DAP12 to mediate immune cell activation by Src, Syk, and Bruton’s tyrosine kinase (Btk) intrinsic signaling^175^ and enhance phagocytosis via the IgG-mediated FcR pathway (Fig. 4b).^176^ The expression of MAC-1 on macrophages is necessary for SLAMF7-dependent phagocytosis of cancer cells.^175^ Whether SLAMF7 is required for CD47-mediated phagocytosis is controversial. Chen et al. showed that during the CD47-SIRPα axis blockade, the phagocytosis of hematopoietic tumor cells was rigidly dependent on SLAMF7,^175^ but He et al. reported that SLAMF7 is not required for CD47-mediated phagocytosis.^177^ Given these controversial research results, the role of SLAMF7 in macrophage phagocytosis requires further investigation.

## Signal pathways of phagocytosis checkpoints

### The CD47-SIRPα signaling pathway

#### The mechanism of the CD47-SIRPα pathway

**Fig. 5:**
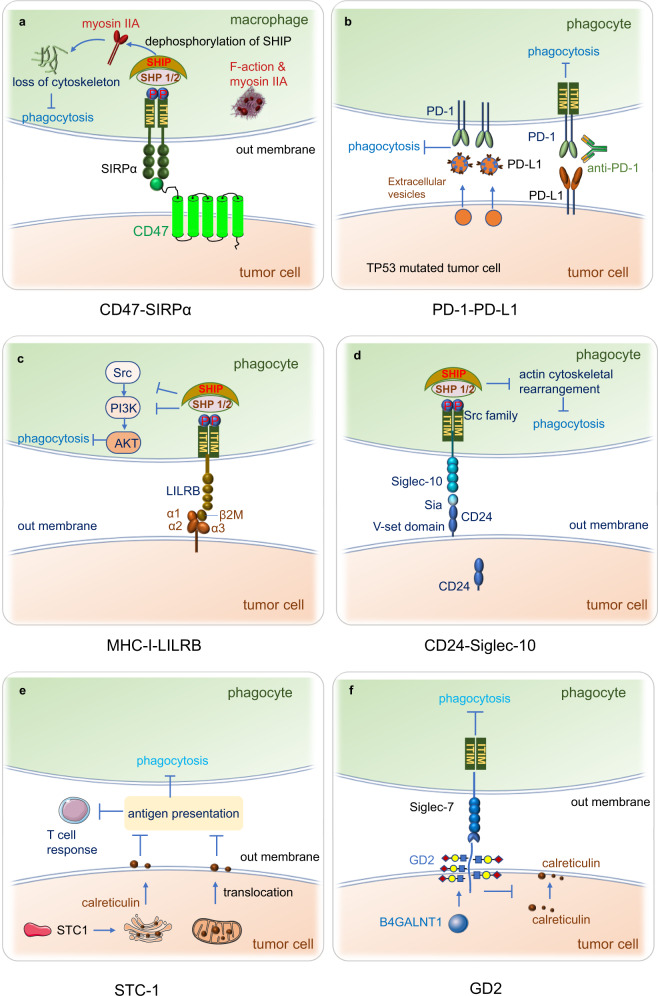
Mechanisms of phagocytosis checkpoints. **a** CD47 on the surface of tumor cells binds to SIPRα on the membrane of the macrophage. This interaction promotes the phosphorylation of ITIM in SIRPα by Src family kinases SHP-1 and SHP-2. The recruitment of SHP-1 and SHP-2 phosphorylate myosin IIA, then suppresses the function of non-muscle myosin IIA, upon dephosphorylation of Myosin IIA in macrophages, the de-polymerization of actin occurs, resulting in the limitation of phagocytosis. **b** TP53 mutation increases the expression of PD-L1 on extracellular vesicles, leading to the block of phagocytosis of tumor cells by macrophages. **c** The β2M of MHC-1 binds to the extracellular region of LILRB1 to form a complex with the MHC- I heavy chain, this novel inhibitory MHC-I-LILRB1 axis inhibits the innate immune system. **d** The inhibitory receptor Siglec-10 on the macrophage surface binds to its ligand CD24 on cancer cells, resulting in an ITIM or ITIM-like motif in the cytoplasmic domain of Siglec-10 combing with Src family kinases. Then Src family kinases phosphorylate ITIM tyrosine in the cytoplasm, then recruit SHP-1/ SHP-2. SHP-1 can specifically bind to the intracellular phosphorylated ITIM domain to dephosphorylate it, leading to cytoskeleton remodeling and phagocytosis inhibition. **e** STC-1 interacts with the “eat me” signal calreticulin and abrogates the membrane calreticulin-directed phagocytosis by macrophages, thus impairing the antigen presentation from macrophages to T cells. Tumor STC-1 is crucial for intrinsic tumor resistance to tumor immunity, it traps calreticulin in mitochondria and ER to inhibit macrophage function and facilitate the tumor cell immune evasion and immunotherapy resistance. **f** GD2 (generated by the enzyme B4GALNT1) binding the Siglec-7 (the inhibitory immunoreceptor) on phagocyte triggers “don’t eat me” signals in the macrophages, calreticulin is an “eat me” signal on the surface of tumor cells, the ligation of GD2 leads to the upregulation of calreticulin, indicating GD2 may inhibit calreticulin signaling

The intracellular region of SIRPα contains an ITIM, which is crucial for the inhibitory activity of the receptor.^178,179^ When an ITAM-containing receptor is triggered, the ITIM-containing receptor SIRPα counteracts cellular activation. The inhibition of this signaling pathway by SIRPα requires tyrosine residues’ phosphorylation in cytoplasmic ITIM sequences, which then recruits and activates the SH2-domain-containing protein tyrosine phosphatases SHP-1 and SHP-2.^178,179^ The recruitment of SHP-1 and SHP-2 phosphorylates myosin IIA and suppresses nonmuscle myosin IIA, which regulates phagolysosomal biogenesis in macrophages and functions in phagocytosis. Upon dephosphorylation of myosin IIA in macrophages, depolymerization of actin occurs, leading to a reduction in phagocytosis^38,180^ (Fig. 5a). The binding of CD47 on tumor cells and SIRPα on phagocytes promotes the phosphorylation of the ITIM in SIRPα by the Src family kinases SHP-1 and SHP-2 and thus contributes to the reduction of phagocytosis.^181^

#### The function of the CD47-SIRPα pathway

**Fig. 6:**
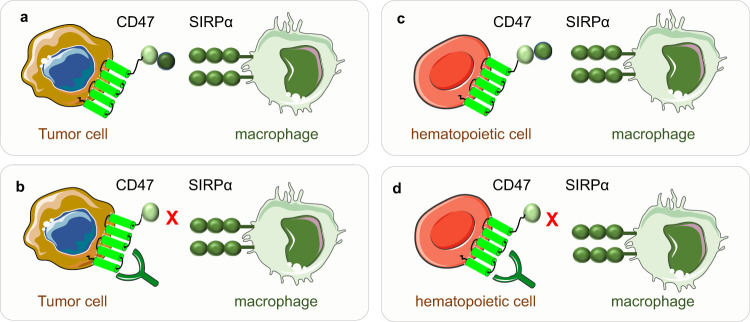
CD47-SIRPα pathway. **a** CD47 expressed on tumor cells interacts with SIRPα expressed on macrophages and other phagocytes to avoid immune surveillance. **b** Targeting CD47 or blocking the CD47-SIRPα axis interrupts their interaction and allows macrophages to phagocyte tumor cells. **c** CD47 expressed on hematopoietic cells or red blood cells interacts with SIRPα expressed on macrophages and other phagocytes to avoid phagocytosis. **d** Targeting CD47 or interrupting the CD47-SIRPα axis allows macrophages to phagocyte hematopoietic cells and thus brings the side effects such as anemia

The best-studied function of CD47-SIRPα is the induction of tumor immune evasion during cancer immunotherapy (Fig. 6a, b). Cancer cells express CD47 highly, which binds to SIRPα on phagocytes, leading to the evasion from immune surveillance. CD47 inhibits the phagocytic function of macrophages, stimulates cell‒cell fusion, activates T cells and affects the migration of neutrophils.^22,23,182–184^ Moreover, CD47 is expressed highly on young RBCs and hematopoietic stem cells (HSCs) to protect them from phagocytosis,^22^ and damaged and senescent RBCs are phagocytosed by macrophages because their expression of CD47 is lower than that in younger RBCs. Targeting CD47 or inhibiting CD47-SIRPα signaling allows macrophages to engulf HSCs and RBCs (Fig. 6c, d). Besides its role in bulk tumor cells, CD47 also plays a crucial role in cancer stemness maintenance and the immunoresistance in cancer stem cells (CSCs).^185^ Furthermore, the CD47-SIRPα interaction also activates the Hedgehog/smoothened (SMO)/GLI family zinc finger 1 (Gli1) pathway in mesenchymal stem cell (MSC)-treated livers after ischemia/reperfusion (IR) stress, and activation of this pathway regulates cell growth, differentiation, and immune function.^186^

### The mechanism and function of the PD-1-PD-L1 axis in phagocytosis

As a T-cell immune checkpoint, the function of the PD-1-PD-L1 axis in T cells has been well elucidated; however, recent studies have shown that this axis also functions in the regulation of the phagocytic ability of TAMs.^9^ PD-1 is expressed not only in T cells in peripheral tissues but also in B cells, activated monocytes, DCs and NK cells.^187,188^ TAMs express high levels of PD-1 compared to splenic macrophages or circulating monocytes, and PD-1 expression increases with tumor volume after engraftment. Furthermore, PD-1 tends to promote the polarization of macrophages to M2 polarization, most PD-1^+^ TAMs are M2-like macrophages, which are regarded as the protumor population in the tumor microenvironment (TME).^9,19,189^ Bone marrow transplantation experiment shows that most PD-1^+^ TAMs originate from circulating leukocytes rather but not resident immune cells.^9^ And PD-1^+^ TAMs show a reduced capacity for phagocytosis in contrast to PD-1^-^TAMs, indicating that PD-1 on TAMs inhibits phagocytosis. PD-L1 deficiency increases phagocytosis by PD-1^+^ macrophages significantly but has no effect on phagocytosis by PD-1^−^ macrophages. Blocking PD-1-PD-L1 signaling with either an anti-PD-1 blocker or a PD-L1 inhibitor (HAC, an engineered small protein lacking an Fc domain to eliminate interference with Fc-mediated phagocytosis) increases macrophage phagocytosis and increases the survival rate of NOD SCID gamma (NSG) mice lacking T cells, indicating the antitumor role of the PD-1-PD-L1 phagocytosis checkpoint.^9^ In addition, TP53-mutated tumor cells secrete more extracellular vesicles and show impaired macrophage phagocytosis, but blocking PD-L1 on the extracellular surface of TP53-mutant cells was able to restore the phagocytic capacity of macrophages, suggesting that the important role of PD-1-PD-L1 is in macrophage phagocytosis in TP53-mutated tumors (Fig. 6b.^190^ The PD-1-PD-L1 axis has direct effects on macrophages in tumors. This evidence implies that PD-1 inhibits not only cytotoxic T-cell activity but also macrophage phagocytosis, revealing a new mechanism of the PD-1-PD-L1 axis in macrophage-mediated phagocytosis. Furthermore, LPS stimulation of TLR4 signaling upregulates PD-1 in macrophages. Ligation of PD-1 in macrophages by PD-L1 potentiates the polarization of tolerogenic STAT6-dependent macrophages and subsequent tumor growth.^191^

In T cells, the tyrosines of the ITIM and the immune receptor tyrosine-based switch motif (ITSM) in the PD-1 intracellular domain are phosphorylated after PD-1 binding to its ligand, thereby recruiting the SH2 domain-containing tyrosine phosphatases SHP-1 and SHP-2 and downregulating TCR signaling to inhibit T-cell activation and proliferation.^192^ Therefore, PD-1 on macrophages may also trigger immunosuppressive signals to inhibit phagocytosis by macrophages; however, the detailed mechanism needs to be further studied.

### The mechanism and function of the MHC-I–LILRB1 axis

#### The mechanism of the MHC-I–LILRB1 axis

The site of contact between LILRB1 on macrophages and MHC-I on tumor cells is located in the conserved α3 domain and β2M subunit rather than the highly polymorphic α1 and α2 domains of MHC-I and 1st and 2nd Ig domain of LILRB1^10^ (Fig. 5c). LILRB1 contains an extracellular region with four Ig-like structural domains (D1-D4), the transmembrane structural domain, and a cytoplasmic tail containing four ITIMs that recruit SHP-1 tyrosine phosphatases(SHIP),^193^ LILRB1 triggers inhibitory signaling through the ITIM in the long cytoplasmic tail.^194^ Specifically, (1) the binding of LILRB1 and MHC-I results in the phosphorylation of ITIM; (2) then, the phosphorylated ITIM recruits phosphatases SHIP after tyrosine residues are phosphorylated by Src family protein tyrosine kinases.^195^ Two hydrophobic residues are symmetrically located at the N-terminal and C-terminal ends of the phosphorylated tyrosine residues in the ITIM of LILRB1, and they affect the ability of the ITIM to bind phosphatases. (3) The recruitment of SHIPs leads to the inactivation of ITAM tyrosine kinases, thereby inhibiting ITAM recruitment for the Syk/ZAP70 kinase family, leading to the activation of PI3K/AKT. (4) The above process promotes cancer cell proliferation and regulates immune cell function negatively, leading to the inhibition of phagocytosis by macrophages.^196^

#### The function of the MHC and MHC-I–LILRB1 pathways

The functions of MHC-I in organ transplantation were reported first. Later, more functions of MHC-I in immunity were explored. The main function of MHC-I in tumor immunotherapy is antigen presentation and induction of immune responses.

##### Antigen presentation

The basic function of MHC-I is to display antigens to CD8^+^ T cells and activate the acquired immune response. MHC-I binds to and presents endogenous antigenic peptides for recognition by CD8^+^ T cells. MHC-I is delivered to the cell surface by the Golgi apparatus to present tumor-associated peptides to CD8^+^ T cells, and CD8^+^ T cells recognize antigenic peptide fragments through the TCR as peptide-MHC-I complexes on transformed cells. Subsequently, CD8^+^ cells are stimulated to undergo clonal expansion and produce cytokines to enable cytolytic effector activity and the killing of tumor cells with antigen-secretion. Tumor cells have developed strategies such as downregulating MHC-I to inhibit HLA-I antigen expression and function to avoid the recognition and destruction by CD8^+^ T cells.^197^

##### Induction of the immune response

MHC-I protects tumor cells from phagocytosis by macrophages and killing by NK cells by binding to inhibitory receptors on the surface of macrophages and NK cells, respectively. Blocking MHC-I or inhibiting LILRB1 either in vitro or in vivo enhanced phagocytosis of tumor cells by macrophages, and tumor cells expressing β2m prevented phagocytosis by macrophages and enabled evasion of the immune response.^10^ This suggests that the MHC-I-LILRB1 signaling axis functions as an antiphagocytic signal. Tumor cells escape NK-cell killing via MHC-I module expression, which was introduced in the section on MHC-I binding proteins.

### The mechanism and function of the CD24-Siglec-10 axis

#### The mechanism of the CD24-Siglec-10 pathway

The inhibitory receptor Siglec-10 on the macrophage surface binds to its ligand CD24 on cancer cells, resulting in the interaction of an ITIM or ITIM-like motif in the cytoplasmic domain of Siglec-10 with Src family kinases.^198^ Then, Src family kinases phosphorylate the ITIM tyrosine in the cytoplasm, thereby recruiting protein tyrosine phosphatases (PPPs), such as SHP-1 and SHP-2.^199^ SHP-1 specifically binds to the intracellular phosphorylated ITIM domain to dephosphorylate it, leading to cytoskeletal remodeling and phagocytosis inhibition (Fig. 5d). In addition, SHP-1 negatively regulates intracellular signal transduction involving cell adhesion molecules, extracellular matrix factors, hormones, cytokines, and growth factors.^200^ Hence, the interaction of CD24 with Siglec-10 inhibits phagocytosis by macrophages and promotes the immune escape of tumors. Blocking the expression of CD24 on tumor cells or Siglec-10 on macrophages genetically or via an antibody enhances the phagocytosis of macrophages and suppresses tumor growth in vivo.^8,201^

Furthermore, CD24 also binds to Siglec-10 on the surface of other immune cells, including T cells,^202^ DC cells,^87,203^ and NK cells,^204^ to inhibit their functions. The mechanisms are all dependent on Siglec10, which has an ITIM or an ITIM-like motif. The ITIM functions in the immunosuppression and tumor immune escape by blocking TLR-mediated inflammation and activating the following intracellular signaling pathways.^205^

#### The function of the CD24-Siglec-10 pathway

The highly expressed CD24 on tumor cells interacts with Siglec-10 on the surface of macrophages to inhibit phagocytosis by macrophages; thus, tumor cells cannot be cleared via phagocytosis by macrophages.^8,201^ Siglec-10, like other members of the Siglec family, preferentially binds to sialylated CD24 in tumor cells, and the sialylation of CD24 helps tumor cells escape engulfment by macrophages.^8^ The interaction between CD24 on tumor cells and Siglec-10 on NK cells helps tumor cells evade the killing effect of NK cells and promotes tumor immune escape.^204^ When interacting with Siglec-10 on the surface of T cells, CD24 blocks activation of the TCR by inhibiting T-cell receptor-related kinases such as Lck and ZAP-70,^202^ thereby promoting escape from killing by T cells. The binding of CD24 to Siglec-10 on the surface of B cells inhibits BCR-regulated signal transduction and promotes tumor escape. Furthermore, the interaction between CD24 and Siglec10 is involved in complex placental immunosuppressive responses.^94^

### Mechanism of STC-1 in phagocytosis

STC-1 promotes tumor angiogenesis and metastasis by upregulating VEGF in a manner dependent on the activation of the PKCβII and ERK1/2 pathways in cancer cells.^206^ STC-1 has been demonstrated to be an intracellular “eat me” signal inhibitor and and unappreciated phagocytosis checkpoint previously. Mechanistically, STC-1 interacts with the “eat me” signal calreticulin in the cancer cell and abrogates membrane calreticulin-directed phagocytosis by APCs, including macrophages and DCs, thus impairing antigen presentation from APCs to T cells, meanwhile, macrophage phagocytosis of cancer cells is suppressed by this process. Tumor STC-1 is crucial for intrinsic tumor resistance to tumor immunity. It plays an essential role in the tumor immune evasion and immunotherapy resistance by trapping calreticulin in mitochondria and the ER to inhibit macrophage function (Fig. 5e). Targeting STC-1 and its interaction with calreticulin may be an approach to enable patients to be susceptible to cancer immunotherapy. Ovarian cancer cells treated with a neutralizing anti-STC-1 monoclonal antibody exhibit higher apoptosis rates than control cells.^207^ In a mouse model of human lung cancer, targeting STC-1-expressed tumor cells exhibits efficient antitumor effects.^208^

### Mechanism of GD2 in phagocytosis

As a sialic acid-linked glycolipid (a sialoglycan), GD2 may be recognized by sialic acid-binding proteins such as Siglecs. GD2 binds to Siglec-7 specifically instead of other Siglecs in humans. Siglec-7 is an immunosuppressive molecule that contains a cytoplasmic ITIM domain and is present in human macrophages and NK cells.^199^ Anti-GD2 disrupts GD2-Siglec-7 interactions and upregulates calreticulin, an “eat me” signal, promoting phagocytosis (Fig. 5f). Anti-GD2 exhibits synergistic effects with anti-CD47 on phagocytosis. The combination of B6H12 (CD47 antibody) and dinutuximab (GD2 antibody) increases the phagocytosis of neuroblastoma cells by microglia significantly, substantially enhances antitumor responses and extends tumor-free survival in a syngeneic model in NSG mice. Monocytes are responsible for these synergistic responses to anti-GD2/anti-CD47.^12^ GD2 and CD47 blockade enhances macrophage phagocytosis by enhancing “eat me” signals and attenuating “don’t eat me” signals and recruits M1-like macrophages for an antitumor response, with potential for clinical application.^12^

## Function of phagocytosis checkpoints in the immune system

### Function of CD47 in immunity

Immune cells, such as monocytes, macrophages, DCs, T cells, and B cells express CD47. which is critical for both innate and adaptive immune responses. CD47 sends a potent potent “don’t eat me” signal to prevent phagocytosis and functions integrally plays in immune responses and autoimmunity.^209^

#### CD47 expression and function in the innate immune system

The CD47-binding protein SIRPα is expressed on macrophages, and the binding of CD47-SIRPα triggers a “don’t eat me” signal, protecting cancer cells from immune clearance.^210^ Another CD47 binding protein, TSP-1, is also expressed on macrophages, and CD47-TSP-1 contributes to the migration of monocytes and leads to nervous system inflammation and the occurrence of disease.^211^ Moreover, NK cells highly express CD47, which regulates the recruitment, activation and proliferation of NK cells.^212^ As a self-marker of DCs, CD47 not only regulates the activation, quantity, maturity, migration and apoptosis of DCs but also participates in the initiation of immune responses in DCs. The expression of SIRPα on DCs inhibits their phagocytosis,^213^ and blocking the CD47-SIRPα pathway activates DC cells to phagocytize tumor cells.^214^ CD47 inhibits the transformation of immature dendritic cells (iDCs) to mature cells in terms of both phenotype and function.^213^ CD47 also regulates DC migration to lymphatic organs. Under inflammatory conditions, CD47-SIRPα interactions are necessary for skin DC migration.^215^ Furthermore, CD47 expressed on neutrophils regulates their transepithelial migration and adhesion; it associates with leukocyte-specific integrin CD11b/CD18 in neutrophils’ membrane, and its loss results in impaired CD11b/CD18 activation. CD47 also regulates chemotaxis of human neutrophils, as SIRPα regulates neutrophil transmigration in vitro.^183,216^

#### CD47 expression and function in the adaptive immune system

CD47 expressed on T cells regulates the activation, proliferation, differentiation and apoptosis of T cells. CD47 is a costimulatory factor for T-cell activation, and the interaction of CD47 on T cells and SIRPα on DCs induces the activation of T lymphocytes by DCs and promotes the proliferation of T cells.^217^ Meanwhile, CD47 modulates T-cell differentiation by affecting both T cells and APCs. The CD47/TSP-1 interaction or blockade of CD47 induces T-cell apoptosis.^218,219^ CD47 limits TCR signaling and killing of irradiated target cells.^220^ The TSP-1/CD47 interaction inhibits TCR signal transduction and induces active T-cell anergy.^221^ In addition, CD47 expressed on Tregs regulates Treg cell generation, proliferation, and differentiation and contributes to Treg neuroprotection by binding to its receptors SIRPα or TSP-.^222^ Furthermore, CD47 is expressed on B cells and limits antibody-mediated phagocytosis and the growth of B cells.^223,224^ The interaction between CD47 on B cells and SIRPα on macrophages also plays a role in cell‒cell contact between B cells and macrophages, which is important for the differentiation of B lymphocytes.

### The PD-1-PD-L1 phagocytosis checkpoint in immunity

#### The PD-1-PD-L1 phagocytosis checkpoint in innate immunity

PD-L1 expressed on DCs facilitates the migration of DCs from the skin to the lymph nodes and triggers intracellular signaling through the cytoplasmic tail of PD-L1. A mutated cytoplasmic domain of PD-L1 impairs CCR7 signaling, including G protein activation, extracellular signal-regulated kinase (ERK) phosphorylation, and F-actin polymerization.^225^ PD-L1 on DCs also regulates immunotherapy and reduces T-cell activation.^226^

PD-L1 expressed on macrophages exerts constitutive signaling effects, leading to suppressed activation and proliferation of macrophages via inhibition of the mTOR signaling pathway in macrophages. PD-L1^−/−^ macrophages stimulate proliferation and activation, and PD-L1 antibody treatment upregulates the production of costimulatory molecules and spontaneous proinflammatory cytokines.^227^ Mechanistically, PD-L1 blockade upregulates costimulatory molecules’ (CD86 and MHC-II) and the secretion of the proinflammatory cytokines (TNFα and IL-12), consistent with the characteristics of M1-type macrophages.^228,229^ However, under the metabolic reprogramming, PD-L1 promotes M2 polarization via the Erk/Akt/mTOR signaling pathway.^230^ On the other hand, PD-1 regulates macrophage polarization to potentiate the inflammation, and PD-1 knockout promotes macrophage M1 instead of M2 polarization by potentiating STAT1, indicating that PD-1 expression is negatively associated with M1 polarization.^231^ PD-1 on macrophages reduces the phagocytic ability of macrophages for tumors and bacteria,^9,232–234^ suggesting that PD-1 affects tumor immunity by both innate and adaptive immune systems.^9,235^

The PD-1-PD-L1 axis plays important roles in protecting against pathogen infection via innate immunity. PD-1 and PD-L1 are expressed on CD4^+^ T cells and CD14^+^ monocytes but not on CD8^+^ T cells in patients with active tuberculosis infection. Blocking the PD-1-PD-L1 pathway increases the phagocytosis and intracellular killing of pathogens by macrophages,^233^ suggesting that the PD-1-PD-L1 pathway has an inhibitory effect on the function of macrophages in terms of phagocytosis of pathogens.

#### The PD-1-PD-L1 phagocytosis checkpoint in adaptive immunity

PD-L1 ligation of PD-1 limits immunogenic responses in T cells. PD-1 contains conserved ITIMs in its cytoplasmic tail, which recruit downstream phosphatases and attenuate activation signals, acting as an immune inhibitory receptor.^236^ PD-1 maintains immune homeostasis and tolerance to prevent immunopathology under physiological conditions, and PD-1 deficiency leads to autoimmune diseases. The PD-1-PD-L1 axis inhibits T-cell activation through a series of signals, eventually leading to a reduction in the activation of transcription factors, such as nuclear factor of activated T cells (NFAT), activator protein 1 (AP-1), and NF-κB, which are critical for T-cell proliferation, activation, survival and effector functions. Furthermore, PD-1 upregulates transcription factors such as basic leucine zipper transcription factor ATF-like (BATF), which can further antagonize effector transcriptional programs to inhibit T-cell functions.^192^

PD-L1 is also expressed in T cells, and PD-L1 blockade reduces the numbers of effector CD8^+^ T cells during the contraction phase of an immune response. Activated CD8^+^ T cells deficient in PD-L1 are more susceptible to Ca-dependent and Fas ligand-dependent killing by cytotoxic T cells, leading to a lower Bcl-xL. PD-L1 on primed T cells helps effector T cells survive in the contraction phase and thereby elicits optimal protective immunity.^237^ Moreover, PD-L1 deficiency results in increased activation of p38 MAPK, which results in the apoptosis of T-cells, indicating that PD-L1 suppresses p38 MAPK activation to preserve T-cell survival.^238^

### MHC-I expression and function in the immune system

MHC-I is a cell surface recognition element expressed on all somatic cells, including all immune cells. It is primarily involved in T-cell-mediated adaptive immune responses but also functions in the innate immune system.

MHC-I is present on the surface of APCs, including both DCs and macrophages. The APCs load endogenous antigenic peptides in the ER onto MHC-I to form a correctly folded trimeric complex (pMHC/β2m), which is modified post-translationally in the Golgi complex, and finally, the complexes are transported to the cell surface, where they are presented to CD8^+^ T cells;^239^ thus, MHC-I bridges the innate and adaptive immunity via antigen-presenting cells.

MHC-I on DCs binds to TCRs on T cells and regulates T-cell differentiation and maturation. T cells that cannot bind to MHC-I are scheduled for apoptosis.^240^ T cells that pass positive selection should not have a strong affinity for MHC; otherwise, they will easily attack themselves. Therefore, only T cells that can bind to MHC via appropriate TCRs with low affinity successfully enter tissues through the blood circulation and exhibit immune surveillance and immune attack abilities. In addition, T cells and B cells also express MHC-1, but its function is rarely studied.^241^

### Function of CD24 in immunity

CD24 is expressed on the surface of a variety of immune cells, including B cells, T cells, DCs, and neutrophils. CD24 interacts with Siglec10 on the surface of various immune cells to exert an immunosuppressive effect. All the above studies demonstrated that CD24 is a critical molecule in the immune system.

#### CD24 expression and function in the innate immune system

CD24 is expressed in all innate immune cells, such as macrophages and DCs, and its main function is endogenous antigen presentation. CD24 on DCs negatively regulates T-cell homeostatic proliferation.^242^ Moreover, CD24 on DCs interacts with Siglec-10 in humans or Siglec-G in mice on the surface of damaged cells; then, SHP-1 binds to the ITIM of Siglec10 and inhibits the activation of NF-kB, which inhibits the release of HMGB1 in turn and negatively regulates damage-associated molecular patterns.^87^ CD24 on the surface of DCs also interacts with Siglec10 in other cell types, which inhibits host inflammatory and immune responses triggered by damage-related molecules,^87^ but it also allows RNA viruses to evade host immunity.^203^ In addition, CD24 on microglia contributes to the activation and proliferation of pathogenic T cells since the costimulatory activity of microglia is reduced in CD24-deficient mice.^243^

#### CD24 expression and function in the adaptive immune system

CD24 was thought to be a marker of B cells originally; it is present highest on B-cell progenitors and is not expressed on terminally differentiated plasma cells because it disappears as B cells mature.^244^ CD24 knock-out leads to a reduction in the numbers of advanced pre-B cells and immature B cells in the bone marrow.^245^ CD24 on activated B cells serves as a CD4 T-cell costimulator for clonal expansion.^246^

As in B cells, CD24 is expressed on peripheral T cells weakly while present expressed on peripheral T cells highly. The difference in CD24 expression between T cells and B cells is that CD24 is upregulated on activated T cells.^247^ CD24 deficiency and CD28 deficiency synergistically suppress CD4 and CD8 T-cell responses.^248^ In addition, highly expressed CD24 on tumor cells binds to Siglec-10 on the surface of T cells and B cells, inhibiting TCR and BCR-related kinases to block activation of the TCR and BCR and ultimately promote tumor immune escape.

## Phagocytosis checkpoints in diseases and the tumor microenvironment (TME)

### CD47 in diseases and the TME

#### CD47 in cancer and the TME

For many types of malignancies, the low early detection rate is an obstacle to improved cancer control;^249,250^ therefore, efforts to identify novel diagnostic markers are valuable.^251^ CD47 has been demonstrated as a diagnostic biomarker for a variety of cancers. It is an innate immune checkpoint and is closely related to the survival in different cancers. High expression of CD47 contributes to tumor cell proliferation and tumor metastasis.

CD47 is overexpressed in a host of hematological malignancies, and its interaction with SIRPα on phagocytes prevents phagocytosis of tumor cells and promotes tumor evasion of immune surveillance.^209,252^ CD47 is expressed highly in both small cell lung cancer (SCLC) and non-small cell lung cancer (NSCLC). In EGFR-mutant NSCLC, the augmented CD47 expression is closely related to the off-target resistance to the tyrosine kinase inhibitor (TKI) gefitinib.^253^ In glioblastoma multiforme (GBM), GBM cells with higher CD47 expression possess the characteristics of stem cells and have poor clinical results,^254^ and irradiation or temozolomide (TMZ) significantly enhances anti-CD47-mediated phagocytosis of GBM cells in vivo and in vitro. Specific inhibition of the TSP-1/CD47 interaction with a peptide antagonist decreases GBM cell invasion.^255^ CD47 is also expressed highly in ovarian cancer, HCC, cholangiocarcinoma (CCA), etc. High expression of CD47 may contribute to the resistance of CSCs to chemotherapy.^256^ In HER2-expressing cells, CD47 is upregulated preferentially, and the interaction between CD47 and HER2 is reflected in the significant difference in the expression levels of CD47 in HER2^+^ versus HER2^−^ breast cancer cells.

#### CD47 in the TME

The TME affects immunotherapy efficacy and patient outcomes in various types of cancer. CD47 functions in immune homeostasis related to cancer prognosis, and its expression is closely related to immune infiltration. TAMs are key components in the TME that participate in the regulation of various biological behaviors and influence tumor growth and progression.^257–259^ Their phagocytic function has been demonstrated to be a key determinant of tumor metastasis and is closely related to the TME.^260^ The blockade of CD47 or SIRPα with blocking antibodies increases the phagocytic activity of TAMs and decreases tumor growth in different tumor models, including models of glioblastoma,^261^ melanoma,^262^ lymphoma,^263^ breast cancer,^264^ and colorectal cancer.^265^ Blocking CD47 promotes antitumor immunity through CD103 + DC-NK cell axis in murine HCC model.^266^ CD47 may also induce T-cell exhaustion by working with T-cell exhaustion markers such as PD-1 and CTLA-4, thus remodeling the TME.^267^

TSP-1, the binding protein of CD47, restricts antitumor immunity via CD47-dependent regulation of innate and adaptive immune cells by regulating angiogenesis and perfusion of the tumor vasculature. Moreover, The TSP-1/CD47 expression and interaction increase under hypoxia to promote tumor growth.^268^

#### CD47 in other diseases

In addition to playing critical roles in cancer and the TME, CD47 also functions in many other diseases. For example, in pulmonary arterial hypertension (PAH), the levels of both CD47 and TSP-1 are increased and promote hypoxia and ROS production in the environment.^269^ In addition, activated CD47 promotes acute kidney injury (AKI) by limiting autophagy, and CD47 has been demonstrated to be a target for preserving renal function following injury.^270^ TSP-1 expression is increased in response to AKI, and blocking TSP-1-CD47 signaling restricts tissue injury caused by ischemic stress in tissues.^271,272^ Furthermore, targeting CD47 attenuates fibrosis induced by various diseases. CD47 mediates immune escape in infectious diseases caused by parasites, bacteria, and viruses, including SARS-CoV-2 in COVID pathogenesis,^273^ and it interferes with the host immune response by binding to SIRPα on immune cells. The disruption of CD47-SIRPα increases the phagocytosis of *P. falciparum*-infected RBCs.^274^

### PD-L1 in diseases and the TME

#### PD-L1 in cancer and the TME

Most tumors, including solid tumors such as melanoma, clear cell carcinoma, NSCLC, and breast cancer, as well as hematological tumors,^65,275^ overexpress PD-L1, whose expression is closely associated with poor prognosis. Tumors evade immune clearance by suppressing T-cell activation via overexpression of PD-L1. Under normal physiological conditions, the PD-1-PD-L1 interaction maintains T-cell immune homeostasis, thereby preventing T-cell hyperactivation and avoiding autoimmune diseases.^59^ However, tumors use PD-1-PD-L1 checkpoint inhibitory signals to evade the immune system, mainly by upregulating PD-L1 expression to suppress T cells, leading to T-cell inactivation and triggering T-cell dysfunction.^66^ High expression of PD-L1 not only inhibits the activity of T cells but also inhibits the activities of APCs such as DCs and macrophages.^9,276,277^ PD-L1 expression on APCs plays an immunosuppressive role in the TME. APCs with PD-L1 expression play a dominant role in the regulation of T-cell immunity and the response to cancer immunotherapy in the context of cancer.^278,279^ On the other hand, PD-1 on macrophages inhibits the phagocytosis of tumor cells, and PD-1-positive TAMs are associated with a reduction in 5-year overall survival in the context of cancer.^9,235^ Therefore, the PD-1-PD-L1 axis interacts with both the innate and adaptive immune systems in the TME.

#### PD-L1 in other diseases

The PD-1-PD-L1 axis plays important roles in many other diseases, such as autoimmune diabetes, rheumatoid arthritis,^280,281^ allergic disease,^282,283^ and neurological disorders. PD-L1 participates in the progression of hypoxia-induced multiple organ injuries, such as injury caused by ischemic stroke, AKI, and obstructive sleep apnea.^284^ Hypoxia upregulates PD-L1 expression via HIF-1α, and PD-L1 is overexpressed in the spleen and central nervous system (CNS) post-stroke. The overexpression of PD-L1 in microglia reduces acute ischemic brain injury by reducing T-cell infiltration and cytokine release. Previous studies have stressed PD-1-PD-L1 as a T-cell checkpoint; therefore, we mainly focused on the functions of these factors in phagocytosis.

The PD-1-PD-L1 axis also plays a critical role in preventing pathogen infection. Sepsis is an overwhelming reaction to infection, and PD-1 on macrophages/monocytes was obviously upregulated during sepsis, together with macrophage dysfunction. The phagocytic function of macrophages during sepsis relies on their PD-1 expression, indicating the role of the PD-1-PD-L1 axis as a phagocytosis checkpoint in microbial clearance.^232^ Furthermore, PD-L1 expression is upregulated on synovial fluid myeloid DCs, T cells and macrophages in rheumatoid arthritis due to the high levels of IFN-γ and TNF-α in RA-derived synovial fluid.^280,281^

### MHC-I in diseases and the TME

#### MHC-I in cancer and the TME

MHC-I on tumor cells interacts with the relevant receptors on almost all immune cells in the TME, thereby affecting tumor immune escape. Downregulation of MHC-I occurs in 40–90% of human tumors and is significantly correlated with poor prognosis.^285^ In contrast, due to irreversible changes in MHC-I expression in tumors caused by genetic mutations, tumors may temporarily upregulate MHC-I to escape natural immune attacks, such as killing by NK cells^286^ and phagocytosis by macrophages.^10^

After anti-CD47 treatment, tumor cells with MHC-I high are more resistant to phagocytosis by macrophages than those with MHC-I low expression. When epithelial cell adhesion molecule or EGFR blockers were used, CD47 and MHC-I double-negative cells were more vulnerable to phagocytosis, whereas the expression of either MHC-I or CD47 alone attenuated macrophage attack, and macrophage resistance was strongest in both double-positive cells. All the above results suggest that MHC-I and CD47 are two independent antiphagocytic signals.^10^

HLA-G is a nonclassical MHC-I, unlike classical MHC, and it is characterized by a low polymorphism rate and tolerogenic function. HLA-G has three soluble isoforms (HLA-G5, HLA-G6 and HLA-G7) that are secreted into the tumor microenvironment and directly inhibit the activation of immune cells.^287^ The expression of HLA-G in solid tumors predicts poor prognosis. However, increased plasma levels of soluble HLA-G in B-cell malignancies are not related with poor clinical outcomes. As an inhibitor of B-cell growth, HLA-G probably exerts an inhibitory effect on tumor growth by interacting with LILRB1, suggesting that HLA-G-LILRB1 axis can be applied to the treatment of B-cell malignancies.^288^

#### MHC-I in other diseases

MHC-I plays critical roles in transplantation, autoimmune diseases and virus infection.^289^ MHC are the main antigens that induce the rejection of allogeneic transplants. The higher the similarity of MHC is between the donor and the recipient, the higher the success rate after transplantation. An HLA match test between the donor and the recipient is required before transplantation.^290^ Regarding autoimmune diseases, more than 50 human diseases have been demonstrated to be related to HLA. For example, high HLA-B27 contributes to the development of ankylosing spondylitis.^291^ Other diseases associated with specific MHC molecules include multiple sclerosis,^292^ Crohn’s disease,^293^ and rheumatoid arthritis.^294^ In addition, MHC-I-restricted CTLs are important effector cells against viral infection, and during symbiosis of the virus and host, the virus escapes elimination and clearance by the host by interfering with the killing activity of CTLs through different pathways.^289^ This process inhibits viral peptides expression by MHC and the recognition of MHC-mutant peptide complexes by the TCR. The function of MHC-I in other diseases related to phagocytosis requires further investigation.

### CD24 in diseases and the TME

#### CD24 in cancers and the TME

CD24 is overexpressed in many cancers, including B-cell lymphomas, gliomas, SCLC, HCC, and breast cancer, and appears to be oncogenic.^295^ CD24 has been demonstrated to be a marker for cancer diagnosis and prognosis. High expression of CD24 on tumor cells not only facilitates tumor progression by affecting the proliferation and migration of tumor cells but also allows tumor cells to escape killing by immune cells via interactions with immune cells around the tumor. When CD24 on tumor cells binds to Siglec-10 on different immune cells, it causes immune cell inhibitory signaling cascades mediated by SHP-1/SHP-2, promoting escape from killing by T and NK cells and engulfment by macrophages.

#### CD24 in other diseases

In addition to its functions in cancers, CD24 plays critical roles in autoimmune diseases, inflammation, and metabolic disorders.

Regarding autoimmune diseases, CD24 polymorphisms are related to the progression and risk of multiple sclerosis,^296^ rheumatoid arthritis.^297^ Mice without CD24 are highly resistant to autoimmune encephalomyelitis experimental.^95^ The detailed role of CD24 in the regulation of autoimmune disease requires further investigation.

Inflammation is involved in many diseases, such as infection, sepsis, liver injury, and chronic graft-versus-host disease; it is the innate immune response to pathogen infection and tissue damage, and CD24 is able to differentiate between DAMPs and pathogen-associated molecular patterns during inflammation.^87^ It selectively inhibits the host response to tissue injury via interaction with Siglec G (mouse) or Siglec10 (human). Regarding metabolic diseases, CD24 binding to Siglec-E is a key inhibitor of obesity-related metabolic dysfunction.^298^

### STC-1 in diseases and the TME

#### STC-1 in cancer and the TME

STC-1 is expressed highly in a variety of cancers, such as colon cancer,^299^ gastric cancer (GC),^206^ ovarian cancer,^299^ breast cancer,^114^ bladder cancer,^300^ glioblastoma,^301^ acute leukemia,^302^ and hepatocellular carcinoma, and higher expression of STC-1 relates to metastasis, lower survival rate and faster progression. Serum STC-1 serves as a promising tumor marker in GC and ovarian cancer because its expression is higher in such patients than in patients with benign tumors,^303,304^ and STC-1 is a potentially useful blood marker for predicting tumor progression and invasion in patients with GC.^305^ STC-1 overexpression increases proliferation, migration, and colony formation in cancer cells. Mechanistically, STC-1 on cancer-associated fibroblasts (CAFs) increases migration and invasion.^299,306^ STC-1 promotes tumor angiogenesis by upregulating VEGF and promoting gastric tumor growth^206^ and promotes cancer cell proliferation, migration and invasion during hypoxia via Bcl-2.^307^ Moreover, STC-1 promotes lipid metabolism and resistance to cisplatin via regulating the FOXC2/ITGB6 pathway in ovarian cancer.^308^ However, in cervical cancer, STC-1 inhibits cell proliferation and invasion and promotes apoptosis.^309^ The expression of STC-1 can be induced under hypoxia by HIF-1 in human cancer cells.^310,311^

#### STC-1 in other diseases

In contrast to its function in most cancers, high expression of STC-1 in other diseases promotes survival. STC-1 overexpression alleviates oxidative stress-induced injury by inhibiting ROS through the mitochondrial pathway^312^ and reduces neuroinflammation; STC-1 overexpression also ameliorates cognitive function by inhibiting the ERK1/2 signaling pathway.^313^ In diabetic nephropathy, STC-1 improves renal injury by inhibiting BNIP3 via the AMPK/SIRT3 signaling, and patients with high levels of STC-1 have a better prognosis.^118^ Serum STC-1 expression is decreased in asthma patients compared with healthy donors, and STC-1 reduces airway hyperresponsiveness (AHR) and inflammation.^314^ Furthermore, the STC-1 concentration in the cerebrospinal fluid was reduced in a heterogeneous group of dementias other than Alzheimer’s disease, especially dementia with Lewy bodies and vascular dementia,^315^ and it also ameliorated cognitive function by inhibiting the ERK1/2 signaling pathway.^313^ Consistently, low expression of STC-1 results in poor prognosis, and NF-κB upregulates miR-155-5p to inhibit STC-1 expression, leading to hepatic mitochondrial dysfunction in nonalcoholic fatty liver and thereby stimulating the occurrence of nonalcoholic fatty liver disease.^316^

### GD2 in diseases and the TME

#### GD2 in cancer and the TME

GD2 has limited expression in normal tissues but is overexpressed on tumors, including gliomas,^317^ melanoma,^318^ osteosarcoma,^319,320^ and soft tissue sarcoma.^321^ The GD2 antibody showed therapeutic effects in all tumors, indicating that GD2 is a promising therapeutic target.

In human neuroblastoma cells, GD2 is the major ganglioside, and progression-free survival was inversely related to circulating GD2 levels, indicating that neuroblastoma tumor gangliosides play a role in accelerating tumor progression.^322^ However, GD2 levels do not seem to correlate with tumor grade,^323^ and GD2 is expressed higher in SCLC than in NSCLC or normal lung cells.^324,325^ In addition, GD2 in melanoma cells is involved in their attachment to extracellular matrix proteins.^326,327^ Antibodies targeting GD2 cause regression of cutaneous metastatic melanoma, and GD2-specific CAR-T cells have antimelanoma activity.^328^

#### GD2 in other diseases

GD2 is most abundant in the central nervous system and modulates the activity of Ca^2+^ channels and transporters. Mice without complex gangliosides (GM2/GD2 synthase knockout) have impaired Ca^2+^ regulation after neuronal development.^329^ Deficiency in neuropathies such as balance, coordination, strength and reflexes develop significantly, indicating the role of GD2 in the maintenance of normal neural physiology.^129^ GM2/GD2 synthase knockout mice also exhibit morphological changes in synaptic vesicles and the mode of synaptic contact with central terminals and deficits in cognitive function and hippocampal plasticity.^330,331^ Mutations of B4GALNT1, which encodes GM2/GD2 synthase, are associated with limb spasticity, dysarthria, peripheral neuropathy, and severe intellectual disability.^332,333^ In addition, the neural ganglioside GD2 is a marker expressed by mesenchymal stem cells (MSCs) isolated from either bone marrow or umbilical cord blood, suggesting that GD2 functions in maintaining stem cell viability.^334^

The expression and working mechanisms of all the reported phagocytosis checkpoints in various diseases are shown in Table 1).

## Targeting phagocytosis checkpoints and clinical applications

### Targeting CD47 and its clinical applications

#### Targeting CD47 in cancer immunotherapy

The role of CD47-SIRPα as an immune checkpoint signaling pathway has been reviewed elsewhere. CD47 is expressed highly on a variety of cancer cells and functions as a key antiphagocytic protein which maintains tumor cells’ resistance to host immune surveillance. The CD47-SIRPα pathway is a phagocytosis checkpoint in macrophages and other innate immune cells, and CD47 has been verified to be a promising therapeutic target due to its antiphagocytic function in tumor cells. Targeting CD47-SIRPα not only disrupts the binding of CD47 and SIRPα and potentiates the phagocytosis ability of cancer cells by stimulating macrophage cytokine secretion and thus stimulating the patients’ immune system^7,335^ but also kills tumor cells through the NK-cell-mediated ADCC effect^336^ and even directly induces tumor cell apoptosis.^337,338^ Moreover, targeting CD47 also enables DCs to phagocytize tumor cells, present the tumor antigen to T cells and activates the adaptive immunity.

#### Development and clinical applications of CD47 antibodies

CD47 was originally discovered as a missing antigen in Rh-negative RBCs by the antibody 1D8 in 1987,^21^ and later, it was defined as an antigen recognized by BRIC126, CIKM1 or BRIC125 monoclonal antibodies.^339,340^ The development of antibodies targeting CD47 has continued since the discovery of CD47. CD47 antibody therapy is mainly divided into three research directions: single drugs, combined therapy with antibody drugs and combined therapy with T-cell checkpoint inhibitors.

Hu5F9-G4 was the 1st humanized antibody targeting CD47, and it was initially used in children with malignant primary brain tumors.^261^ Hu5F9 has a curative effect against five different childhood brain tumors. It can be used to treat a variety of malignant tumors of the central nervous system. Another antibody, SIRPαD1-Fc, a novel CD47-targeting fusion protein, increases the autophagy of NSCLC cells by inactivating the Akt/mTOR pathway and increasing ROS levels Table 1.

**Table 1:** Phagocytosis checkpoints expression and working mechanisms in various diseases

Checkpoint	Disease	Expression and mechanisms	Reference
CD47	diffuse large B-cell lymphoma	highly expressed, immune evasion via CD47-SIRPα axis	^391^
CD47	chronic lymphocytic leukemia	overexpressed, targeting CD47 promotes apoptosis	^392^
CD47	Burkitt lymphoma	overexpressed	^263,393^
CD47	primary effusion lymphoma	highly expressed, promotes phagocytosis	^394^
CD47	T-cell lymphoma	variably expressed. CD47 promotes TCL metastasis by up-regulating AKAP13-mediated RhoA activation	^395,396^
CD47	acute myeloid leukemia	highly expressed, increase leukemia stem cells	^397^
CD47	myelodysplastic syndrome	The higher CD47 indicates poor prognosis in MDS patients.	^398^
CD47	multiple myeloma	High CD47 expression in MM patients is related to p53 deletions and elevated β-2 macroglobulin levels.	^399,400^
CD47	SCLC	highly expressed	^401^
CD47	NSCLC	decreasing neutrophil apoptosis and phagocytosis in NSCLC	^253^
CD47	glioblastoma	GBM cells with higher CD47 expression possess the characteristics of stem cells and have poor clinical results	^402^
CD47	breast cancer	Highly expressed CD47 promotes CSC resistance.	^403^
CD47	ovarian cancer	CD47 inhibits macrophage phagocytosis and promotes cell growth and metastasis in EAOC.	^404^
CD47	hepatocellular carcinoma	Macrophages induce CD47 upregulation in HCC patients by IL-6 and correlate with poor survival.	^46^
CD47	cholangiocarcinoma	Blocking CD47-SIRPα pathway increases phagocytosis of macrophages and inhibits CCA growth and metastasis.	^405^
CD47	pulmonary arterial hypertension	The activation of CD47 by TSP-1 promotes hypoxic PAH and the activated CD47 inhibits the upregulation of Cav-1 and promotes ROS in PAH.	^269^
CD47	systemic lupus erythematosus	CD47 has been demonstrated to potentiate the inflammatory response in SLE patients.	^406,407^
CD47	acute kidney injury	The activated CD47 promotes AKI by limiting autophagy.	^270–272,408^
CD47	Ischemia-reperfusion injury	under hypoxia and following IR, TSP-1/CD47 axis is induced in renal tubular epithelial cells (RTEC).	^271^
CD47	cerebral malaria	The lymphocytes increased in CD47-blocked mice, and the IL22, TNF-α, and IFN-γ were increased in the circulation.	^409^
CD47	fibrosis	Targeting CD47 attenuates fibrosis induced by various diseases.	^410^
CD47	COVID-19 pathogenesis	CD47 mediates the immune escape in infectious diseases caused by parasites, bacteria, and viruses.	^411,412^
CD47	colon cancer	Inhibition of CD47 reduced the migration of SW480 cells.	^413,414^
CD47	autoimmune uveitis	CD47 regulates the SIRPα+ on DCs, which is crucial to the induction in EAU.	^415^
CD47	uveal melanoma	Patients with higher CD47 have higher CD4+ and CD8+ T cells.	^283^
CD47	thyroid cancer	CD47 involves the up-regulation of the PD-1 oncogenic signaling	^416^
CD47	type1 diabetes	CD47-SIRPα deficiency or interruption leads to the upregulation of lymphocyte activation, β-cell destruction and cytotoxicity.	^417^
CD47	age-related macular degeneration	Pharmacological activation of CD47 induces the resolution of subretinal chronic inflammation that leads to irreversible blindness in AMD.	^418^
CD47	isoproterenol (ISO)-induced cardiac hypertrophy	Blocking CD47 inhibits isoproterenol-induced cardiac hypertrophy via autophagy	^419,420^
CD47	atherosclerosis	CD47-inhibition restores phagocytosis and prevents atherosclerosis	^421^
CD47	pancreatic cancer	The restoration of miR-340 reduces *CD47* and facilitates phagocytosis, suppressing tumor progression	^422^
PD-L1	autoimmune diabetes	In pancreatic beta cells in NOD mice, the increase of PD-L1 suppressed disease progression	^423^
PD-L1	inflammatory bowel disease	PD-L1 was expressed highly in intestinal epithelial cells of inflammatory bowel disease (IBD) patients	^424^
PD-L1	allergic asthma	Upregulation of PD-L1 downregulates AHR and inflammation.	^46,282^
PD-L1	multiple sclerosis	Increased PD-L1 expression inhibits proinflammatory response.	^425,426^
PD-L1	rheumatoid arthritis	Upregulation of PD-L1 inhibits proinflammatory response.	^280,281^
PD-L1	psoriatic	Upregulation of PD-L1 on T-MSCs inhibits and decreases immune response.	^427^
PD-L1	atherosclerotic plaque	PD-1/PD-L1 pathway downregulates the proatherogenic T cell response and atherosclerosis.	^428–430^
PD-L1	renal cell carcinoma	High levels of PD-L1 expression show a worse prognosis.	^431,432^
PD-L1	HCC cancer	Increased expression of PD-L1 was associated with a significantly poorer prognosis.	^433^
PD-L1	NSCLC	Expression of PD-L1 indicates a worse prognosis.	^434,435^
PD-L1	melanoma cancer	PD-L1 correlates with worse patients survival.	^436,437^
PD-L1	breast cancer	PD-L1 relates to tumor size and a lower survival rate	^438^
PD-L1	ovarian cancer	PD-L1-high patients have a poor prognosis	^439^
PD-L1	pancreatic cancer	PD-L1-high patients indicate a poorer prognosis than the PD-L1-low patients.	^440^
PD-L1	cervical cancer	PD-L1 acts as a prognostic factor of poor survival.	^441^
PD-L1	colon cancer	PD-L1 is upregulated and correlates to poor prognosis.	^442,443^
PD-L1	esophageal cancer	PD-L1 is highly expressed and indicates poorer survival.	^444^
PD-L1	large B-cell lymphoma	PD-L1 positive DLBCL patients had a shorter survival rate than those PD-L1 negative patients.	^445^
PD-L1	glioblastoma	PD-L1 high expression correlates to shorter overall survival.	^446^
PD-L1	prostate cancer	PD-L1 was highly expressed in high-risk patients. PD-L1 positivity relates to independent unfavorable prognostic.	^447,448^
PD-L1	gastric cancer	High PD-L1 indicates a shorter survival time.	^449^
GD2	neuroblastoma	Progression-free survival (PFS) was inversely related to circulating GD2 levels	^450^
GD2	SCLC	GD2 is expressed in SCLC lines and GD2 expression is also much higher in SCLC cell lines than in normal lung cell lines	^324,325^
GD2	osteosarcoma	GD2 is highly expressed in osteosarcoma cells.	^319,320^
GD2	ewing sarcoma	The expression of GD2 is a characteristic of Ewing sarcomas. It is a target antigen for immunotherapy.	^451^
GD2	soft tissue sarcoma	A large percentage of soft tissue sarcoma patients express GD2.	^452^
GD2	gliomas	GD2 is a commonly expressed surface antigen of gliomas.	^453^
GD2	Melanoma	Melanoma cells contain abundant amounts of GD2.	^326,327^
CD22	leukemia	CD22 is highly positive in various proportions in leukemia.	^454^
CD22	B-cell lymphoma	CD22 is expressed in non-Hodgkin’s lymphoma, DLBL, and small lymphocytic lymphoma.	^147–149^
STC-1	gastric cancer	STC-1 was upregulated in gastric cancer, and higher expression of STC-1 related to survival rate. STC-1 is a potential blood marker for predicting biological tumor aggressiveness.	^304,305,307^
STC-1	colorectal cancer	STC-1 was higher in the cancer tissue and indicated a poor prognosis.	^455^
STC-1	ovarian cancer	STC-1 expression was upregulated in ovarian cancer patients and it correlated with ovarian cancer patients’ overall survival.	^306,456^
STC-1	breast cancer	STC-1 expression is upregulated and is correlated with poor prognosis.	^457^
STC-1	bladder cancer	The expression of STC-1 was upregulated in a higher stage bladder cancer and the high expression of STC-1 predicts a poor prognosis in bladder cancer.	^300,458^
STC-1	glioblastoma	The STC-1 expression is increased in glioblastoma tissues, and STC-1 revealed a significant association with poor outcomes in patients.	^301^
STC-1	acute leukemia	High STC-1 gene expression is associated with shorter overall survival in acute leukemia.	^302^
STC-1	hepatocellular carcinoma	Higher serum STC-1 level in HCC patients was correlated with poorer survival.	^459^
STC-1	neuroinflammation	STC-1 overexpression reduces brain injury,	^313^
STC-1	diabetic nephropathy	STC-1 improves renal injury in diabetic nephropathy. Patients with high levels of STC-1 have a better prognosis.	^118^
STC-1	asthma	Serum STC-1 is decreased in asthma. STC-1 reduces airway hyperresponsiveness (AHR) and inflammation.	^314^
STC-1	lung injury	STC-1 protects against oxidant-induced lung injury.	^312^
STC-1	nonalcoholic fatty liver	Suppressed STC-1 expression stimulates the occurrence of nonalcoholic fatty liver.	^316^
MHC-I (HLA-B27)	ankylosing spondylitis	HLA-B27 is thought to be important in the pathogenesis of ankylosing spondylitis, contributing approximately 20.1% to the heritability of ankylosing spondylitis, associated with the presentation of non-standard antigenic peptides.	^291^
MHC-I (HLA-B27)	Lyttle’s syndrome	Reiter’s syndrome is an HLA-B27-associated disease.	^397^
MHC-I (HLA-B27)	acute anterior uveitis	HLA-B27-associated immune response promotes the development of acute anterior uveitis.	^460^
MHC-I (HLA-B27)	juvenile rheumatoid arthritis	About 10% of juvenile chronic arthritis patients carry HLA-B27.	^461^
MHC-I	celiac disease	Different HLA alleles play opposite roles in celiac disease.	^462^
MHC-I	Graves’ disease	Patients carrying HLA-A10 and HLA-B8 Graves’ disease tend to develop the disease at an earlier age.	^463^
MHC-I	juvenile diabetes mellitus	Different subtypes of HLA affect juvenile diabetes mellitus onset and progression.	^464^
MHC-I	melanoma	Tumor escapes through MHC expression deficiency.	^465^
MHC-I	Laryngeal carcinomas; colorectal carcinomas; bladder carcinomas	HLA haplotype loss in laryngeal cancer is associated with loss of heterozygosity in the chromosome 6p21 region	^466^
MHC-I	head and neck squamous cell carcinoma	Lower expression of HLA is related to the survival and recurrence of HNSCC.	^467^
MHC-I	breast carcinoma	The loss of heterozygosity on a chromosome 6 (the HLA-I region of chromosome) and 15 (the β2-microglobulin (β2M) region) is highly prevalent in breast cancer.	^468^
MHC-I	neuroblastoma	MHCI expression relates to neuroblastoma stage and prognosis.	^469^
MHC-I	colorectal liver metastases	High expression of MHCI with significantly increased overall survival.	^470^
MHC-I	ovarian cancer	HLA-A02 serves as a prognostic biomarker.	^471^
MHC-I	non-small-cell lung cancer	Reduced HLA expression did not affect prognosis, but the heterogeneous expression of HLA had a poor prognosis.	^472^
CD24	autoimmune disease	CD24 has co-stimulatory activity and furthermore, CD24 is a genetic checkpoint for balanced T cell proliferation.	^473^
CD24	inflammation	CD24 relates to various DAMPs, including nucleolin, heat-shock protein and high mobility histone box protein 1.	^87^
CD24	GvHD	The CD24-Siglec-G/10 selectively modulates the host response to DAMPs and regulates the immune response.	^87^
CD24	bacterial and viral infections, sepsis, COVID-19	CD24 acts as an important immune regulator in complex physiological diseases characterized by excessive inflammation	^474^
CD24	multiple sclerosis	Individuals with the CD24V/V genotype have a greater susceptibility to and progression of MS than individuals with the CD24A/V and CD24A/A genotypes.	^475^
CD24	Breast cancer	CD24(+) cells are responsible for breast tumor heterogeneity.	^476^
CD24	colorectal cancer	CD24-dependent activation of the MAPK pathway promotes colorectal cancer cell proliferation in vitro and in vivo.	^477^
CD24	lung adenocarcinoma, ovarian carcinoma, glioblastoma	CD24 inhibits tissue factor pathway inhibitor-2 (TFPI-2), which in turn promotes tumor cell invasion in a c-Src-dependent manner.	^478^
CD24	osteosarcoma, prostate cancer,	CD24 promotes the infiltration and metastasis of various tumors by HIF1, EPCaM	^479^
CD24	cervical cancer	CD24 is upregulated in cervical cancer tissues and inhibits apoptosis by affecting the MAPK signaling pathway in cervical cancer.	^480^
CD24	hepatocellular carcinoma	High CD24 is associated with poor prognostic markers, and overexpression of CD24 is associated with high proliferation and metastasis.	^481^
CD24	Prostate cancer	CD24 is significantly increased in contrast to prostate hyperplasia.	^482^
CD24	Ovarian Cancer	CD24 expresses not only in the cell membrane but also intracellularly, appearing in exosomes.	^373^

The combination of CD47-SIRPα targeting with other treatments likely achieves better efficacy. Common combination therapies include treatment with other therapeutic antibodies, recruitment of macrophages, combined chemotherapy and radiotherapy and inhibition of tumor metastasis. For example, blocking the CD47-SIRPα pathway together with treatment with sodium stibogluconate (SSG), an antileishmaniasis drug, overcomes the resistance of anti-CD20-opsonized B-cell lymphoma cells to neutrophil killing.^341^ Hu5F9-G4 (now known as magrolimab) is in a phase II/III clinical study for AML and has shown a favorable safety profile in combination with azathioprine (AZA).^342,343^ Magrolimab in combination with AZA demonstrated early efficacy in AML patients with mutated TP53.^344,345^

An increasing number of companies, including overseas companies such as Forty-seven (merged by Gilead in 2020), Celgene, Trillium, Alxoncology and domestic companies such as IMAB and ImmuneOnco, are currently developing drugs targeting CD47, specifically monoclonal antibodies, bispecific antibodies, fusion proteins and small molecules; many of these drugs have entered the clinical research stage, but there are no such drugs on the market yet. In 2019, Forty-seven announced that its CD47 monoclonal antibody magrolimab showed excellent and sustainable clinical efficacy. Since then, a pipeline of research on CD47 has emerged all over the world. The most advanced drugs in development are in phase III clinical trials, and most of the drugs in development are in phase I/II clinical trials; please refer to the detailed list of clinical trials in the USA and China (Table 3).

#### Challenges of targeting CD47

##### Red blood cell toxicity

CD47 is universally expressed on normal cells, including RBCs and T lymphocytes. thus, special attention should be given to whether the developed antibodies have adverse effects on normal cells. Targeting CD47 leads to the phagocytosis of RBCs by macrophages and causes the agglutination of the RBCs, ultimately leading to the lysis of RBCs. In addition, NK cells or macrophages may attack RBCs via Fc-mediated effector function, via either ADCC or antibody-dependent cell-mediated phagocytosis (ADCP). Therefore, avoiding binding with RBCs has become a primary concern in CD47 antibody drug development.

##### T lymphocyte toxicity

CD47 is expressed on T lymphocytes; when a CD47 antibody binds to CD47 on T lymphocytes, it may cause T-cell apoptosis, which may prevent clinical development, as T cells are key immune cells in cancer immunotherapy.

##### Selection of IgG subclasses

The Fc part of the antibody activates FcR on NK cells, macrophages, or neutrophils, leading to tumor cell lysis via ADCC or ADCP. In addition, antibodies directly activate the complement pathway to enable killing of antibody-coated tumor cells via complement-dependent cytotoxicity (CDC).^346,347^ The four subtypes of IgG bind different types of FcR with different binding capabilities and different effector functions in ADCC and ADCP, and the IgG subclass must be taken into consideration during antitumor therapeutic antibody selection.^348–350^ Since most CD47 antibodies preferentially bind to RBCs, if IgG1 is selected, immune cells such as NK cells and macrophages will be activated by RBCs. Therefore, to avoid RBC toxicity caused by CD47 antibodies, the IgG4 subtype was selected for all CD47 antibodies in development, but the antitumor activity of these antibodies is reduced.

##### High blood pressure

The CD47/TSP-1 axis regulates blood pressure,^351^ and CD47 knockout mice have normal central pulse pressure but elevated peripheral blood pressure. Targeting CD47 achieves vasopressor activity to maintain global hemodynamics under stress.^352^

##### Differences in binding affinity between animal models and humans

The selection of an animal model is very important when evaluating CD47 targeting. The binding affinity between CD47 from humans and SIRPα from NSG mice is 10 times higher than that between CD47 and SIRPα from humans, indicating that positive results in mice may not translate to success in human clinical trials. Furthermore, the mice used in the animal model are immuno-comprised animals that lack a complete immune system. Xenotransplantation under ideal conditions warrants further investigation.

#### Proposed strategies and future perspectives for targeting CD47

##### Designing a CD47 antibody with weak binding to RBCs

The high expression of CD47 on RBCs means that RBCs bind to CD47 antibodies preferentially,^353^ but it is still possible to develop antibodies with antitumor activity and without hematological toxicity since the molecular conformation of CD47 on tumor cells is distinct from that of CD47 on RBCs.^354–356^ TJC4 was designed with the above concept and is in a phase II clinical trial currently.^357^

##### Designing a CD47 antibody based on the differences between RBCs and tumor cells

Tumor cells are different from RBCs in terms of both morphology and molecular biology, and it is possible to develop antibodies based on these differences.^358^ AO-176 was designed based on this idea, and it selectively binds to tumor cells rather than RBCs; another antibody, RRX-001, was also designed according to the above concept and does not cause anemia.^359^

##### Targeting SIRPα instead of CD47

Targeting SIRPα can also block the CD47-SIRPα pathway. Since SIRPα is not expressed on RBCs, targeting SIRPα will not cause the depletion of RBCs and platelets. The antibodies ADU-185, TTI-621 and ALX148 were designed with high affinity for SIRPα and low affinity for blood cells.^360–362^

##### Targeting QPCTL-mediated CD47 pyroglutamylation in CD47-SIRPα signaling

**Fig. 7:**
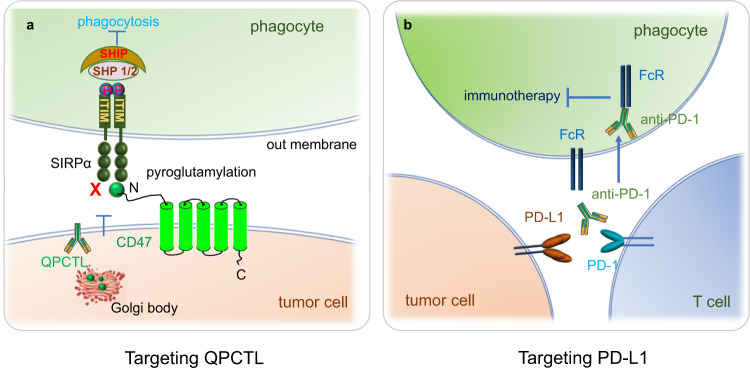
Targeting QPCTL and targeting PD-L1. **a** QPCTL is the key enzyme for pyroglutamylation of CD47. Targeting QPCTL significantly reduces the binding ability of CD47 to SIRPα and prompts phagocytosis of tumor cells by macrophages thus regulating tumor immunity. **b** PD-1 is expressed on T cells, the antibody targeting PD-1 can bind to FcR on phagocytes and then be engulfed by the phagocytes, leading to the inhibition of cancer immunotherapy

The pyroglutamylation of CD47 is essential for the binding between CD47 and SIRPα, and QPCTL is the key enzyme for pyroglytamylation of CD47.^338,363^ Targeting QPCTL significantly attenuates the binding ability of CD47 to SIRPα and increases phagocytosis of tumor cells by macrophages, thus regulating tumor immunity (Fig. 7a),^50,52,364^ and targeting QPCTL avoids anemia since QPCTL is not expressed on mature RBCs.

### Targeting PD-1-PD-L1 in phagocytosis and clinical applications

After the discovery of the important role of PD-1-PD-L1 in tumor immune escape, targeting of this pair has been rapidly applied to clinical treatment. PD-1 and PD-L1 blockade have become important clinical treatments. Targeting the PD-1-PD-L1 immune checkpoint has achieved remarkable results in clinical applications, with unprecedented progress; numerous clinical trials are always ongoing. A PD-1 or PD-L1 antibody blocks the immunosuppressive effect of PD-1-PD-L1 and restores the ability of T cells to kill tumors. Since the approval of nivolumab in 2014, many companies have successfully developed and approved PD-1-PD-L1 antibody drugs. There are many such antibody drugs in the clinic for a variety of different indications.

Immune checkpoint inhibitor drugs, such as PD-1 and PD-L1 antibodies, have multiple effects in immunotherapy. They not only suppress the inhibitory interaction between T cells and tumor cells but also block the binding between macrophages and tumor cells. The combination of CD47 and PD-1 blocking antibodies results in a synergistic ability to inhibit tumor growth, providing a new strategy for immunotherapy.^9^ Recently, bispecific antibodies targeting PD-1-PD-L1 and CD47-SIRPα have also been applied in clinical trials as a new strategy. In addition, TAMs can remove PD-1 blocking antibodies from T cells via FC-FcγR to weaken the immune response (Fig. 7b).^365,366^ Therefore, Fc-engineered IgG variants that disrupt FCR binding and combination therapies with agents that inhibit FcγR binding are better options for immunotherapy.

TAMs, a type of M2-polarized macrophage, eliminate or suppress T-cell-mediated anti-tumor responses. Carfilzomib is an FDA-approved drug for treating patients with relapsed/refractory multiple myeloma, recent research indicated that Carfilzomib induces M2 macrophages to express cytokines secreted by M1 macrophages, and phagocytizes tumor cells, as well as presents antigens to T cells. Mechanistically, treatment of Carfilzomib elicited unfolded protein response (UPR), activated IRE1α to recruit TRAF2, and enhanced NF-κB activation to transcribe genes encoding M1 markers in M2 macrophages, thus leading to enhanced phagocytosis of macrophages.^367^ The combination of Carfilzomib with PD-1 antibody exerts the synergistic effect in lung cancers.^367^

### Targeting MHC-I-LILRB1 and clinical applications

MHC-I expression on tumor cells renders their resistance to phagocytosis, which may be because of the inhibitory interaction between the β2M subunit of MHC-I on cancer cells and LILRB1 on phagocytes. Therefore, targeting the MHC-I-LILRB1 axis may enhance the phagocytosis of tumor cells. HLA-G is rarely expressed in normal cells and mainly found in tumor cells, as a ligand, it interacts with LIRB1 receptor with the highest affinity. Monoclonal antibodies against HLA-G have been successfully used against cancer as part of an immune checkpoint suppression strategy.^368^ Since the most important function of MHC-I is antigen presentation and since MHC-1 is ubiquitously expressed on all types of cells, targeting MHC-I enhances phagocytosis of tumor cells by macrophages but may also lead to the loss of T-cell recognition of antigens and tumor immune evasion. There are currently no MHC-I-targeting drugs on the market.

Although cytotoxic T cells’ antitumor activity is dependent on their interaction with MHC-I, specific blockade of the LILRB1/β2M axis is a potential target for innate immune drugs. LIRB1 is not only present mainly in myeloid cells but also highly expressed in CSCs and probably modulates tumor progression and recurrence directly and determines tumor stem cell activity. Importantly, studies in mice with knockout of the relevant targets showed that LILRB1 does not affect normal development or hematopoiesis. Hence, LILRB1 may be an ideal target for tumor therapy.^369^ Preclinical data show that BND-22 exerts broad antitumor effects by targeting LILRB1-mediated “don’t eat me” signals in macrophages and activating NK and CD8^+^ lymphocytes, effectively inhibiting tumor growth in melanoma and colorectal cancer, prolonging the survival of model mice, and inhibiting the spread of cancer cells. Treatment of melanoma and colorectal cancer with BND-22 prolongs the survival of mice and inhibits the spread of cancer cells.^370^ NGM707 is another novel dual antibody antagonist targeting LIRB1 and LIRB2. Preclinical data suggest that NGM707 stimulates myeloid and lymphocyte activation by blocking LIRB1 and reprograms inhibitory myeloid cells to a stimulatory state by blocking LIRB2.^371^ The HLA-B57-Fc fusion protein iosH2 binds to LILRB1/2 and KIR3DL1 with high affinity and blocks the binding of HLA-G and ANGPTL to LILRB1/2, promoting the conversion of macrophage in the M2 phenotype to the M1 phenotype and thus enhancing phagocytosis of cancer cells in vitro; iosH2 also increases the cytotoxicity of T cells and NK cells in coculture with cancer cell lines.^372^

### Targeting CD24 and clinical applications

CD24 emerges as a potential therapeutic target due to its important role in cancers. Antibodies targeting CD24 have been widely exploited for cancer treatment. Preclinical studies of CD24 antibody-mediated targeted therapy have been reviewed previously,^373^ and there has been no clinical study targeting CD24 to date.

Blocking or reducing the interaction between CD24 and Siglec-10 by reducing CD24 expression via monoclonal antibodies or gene editing potently enhances the phagocytosis of tumor cells with high CD24 expression by macrophages.^8^ Siglec-10 binds to CD24 in a sialic acid-dependent manner. Recent studies have demonstrated that increased tumor sialic acid loss decreases Siglec’s inhibitory effect.^374^ Selective removal of sialic acid from tumor cells using antibody-sialidase conjugates has been verified to significantly enhance tumor cell susceptibility to ADCC and enable immune cell killing of desialylated cancer cells. For example, in sepsis, treatment of CD24 with sialidase abolishes the interaction between Siglec-10 and CD24.^375^

Due to its high expression in cancers and its role as a biomarker of some CSCs and an antiphagocytic checkpoint, CD24 may be a promising target for cancer immunotherapy. Although its expression on immune cells leads to harmful adverse effects, investigators have also attempted to explore the efficacy of anti-CD24-based cancer therapy in preclinical models.

### Targeting GD2 and clinical applications

Tumor-specific GD2 is the first ganglioside that is demonstrated to be an effective target antigen for cancer immunotherapy with monoclonal antibodies or CAR-T cells, and numerous clinical trials are underway (Table 2). Targeting GD2 with mAbs causes dephosphorylation of focal adhesion kinase (FAK) and inhibits activation of the PI3K/Akt pathways, thus inducing apoptosis and attenuating the migration of cancer cells.^376^ The murine antibodies m3F and 14.G2a were developed in the 1980s and demonstrated promising effects in vitro and in vivo in neuroblastoma, which provides a strong rationale for clinical trials.^377^

**Table 2:** Antibodies/drugs/ peptide agonists targeting phagocytosis have not yet been tested in the clinical stage

Name	Mechanism	Target	Disease
B16H12.2	CD47 antibody, it promotes phagocytosis of cancer cells, and induces long-term remissions in the treated mice.	CD47	leukemia
AMMS4-G4	CD47 antibody, it enhances macrophage infiltration and enhances the anti-tumor activity of opsonizing antibody modestly.	CD47	leukemia
ZF1	ZF1 binds CD47 with high affinity, induces robust, and phagocytosis of leukemic cancer cells by macrophage.	CD47	leukemia
ADU-1805	ADU-1805 binds to all known human SIRPα alleles, which shows minimal binding to SIRPβ1, while cross-reacting with SIRPγ, and potently blocking the interaction of SIRPα with CD47.	SIRPα	lymphadenoma
Luteolin	Luteolin binds to isoQC, which attenuates pyroglutamylation of CD47 and abrogates the interaction between CD47 and SIRPα and promotes the macrophage-mediated phagocytosis.	CD47 (isoQC)	multiple myeloma
KWAR23^263^	A blocking mAb to human SIRPα, and it binds SIRPα with high affinity and disrupts its binding to CD47. It can enhance the efficacy of rituximab in human BL but it is inert when used by one its own.	SIRPα	Burkitt’s lymphoma
4N1K/4N1	4N1K is analogous to the C-terminal part of TSP-1, and may bind to receptors independent from CD47.	CD47	AML
PKHB1	A TSP-1-derived CD47 agonist peptide, in T-ALL cell lines, PKHB1 induced caspase-independent and calcium-dependent cell death.	CD47	T-ALL
Nivolumab	Specific PD-1 antibody block PD-1 and PD-L1 interaction, to prevent T cell inactivation, blocking the immune escape of cancer cells and improving the ability of the immune system to kill cancer cells.	PD-1	Metastatic melanoma, Metastatic NSCLC, Renal cell carcinoma (RCC), Classical Hodgkin’s lymphoma, Head and Neck Squamous cell carcinoma (HNSCC), Urothelial Carcinoma Microsoft Himalaya, Esophageal carcinoma
Pembrolizumab		PD-1	Metastatic melanoma, Metastatic NSCLC, Classical Hodgkin’s lymphoma, HNSCC Microsoft Himalaya
Pidilizumab		PD-1	Diffuse large B-cell lymphoma
Toripalimab		PD-1	Metastatic melanoma, Bladder Urothelial Carcinoma, Esophageal carcinoma, Nasipharyngeal carcinoma
Sintilimab		PD-1	Lung squamous cell carcinoma, Liver hepatocellular carcinoma, Non-Small Cell Lung Cancer, Hodgkin’s lymphoma, Esophageal carcinoma, Stomach adenocarcinoma
Camrelizumab		PD-1	Liver hepatocellular carcinoma, Hodgkin’s lymphoma, Esophageal carcinoma, Non-Small Cell Lung Cancer, Advanced asipharyngeal carcinoma, Recurrent and metastatic nasopharyngeal carcinoma, Non-small-cell carcinoma,
Tislelizumab		PD-1	Urothelial Carcinoma, Hodgkin’s lymphoma, Non-Small Cell Lung Cancer, Liver hepatocellular carcinoma, MSI-H /dMMR
Penpulimab		PD-1	Hodgkin’s lymphoma,
Zimberelimab		PD-1	Hodgkin’s lymphoma,
Serplulimab		PD-1	MSI-H solid tumor
Atezolizumab	Specific PD-L1 antibody block PD-1 and PD-L1 interaction, improving host immune killing.	PD-L1	Urothelial carcinoma, Metastatic NSCLC, Hepatocllular carcinoma,
Avelumab		PD-L1	MSI-H /dMMR coloretal cancer and stomach adenocarcinoma
Durvalumab		PD-L1	Non-small-cell carcinoma, Small cell lung cancer
Sugemalimab		PD-L1	Non-small-cell carcinoma,
Dinutuxima	Chimeric antibody specific binds to GD2	GD2	Neuroblastma
Dinutuximab beta	Humanized monoclonal antibody	GD2	Neuroblastoma
Hu3F8 (Naxitamab)	Humanized antibody targeting GD2	GD2	Neuroblastoma
Hu14.18K322A	Humanized antibody targeting GD2	GD2	Neuroblastoma
Moxetumomab pasudotox-tdfk	CD22 antibody fused to truncated pseudomonas exotoxin (PE38). Targeting CD22 for delivery of cytotoxic drugs.	CD22	leukemia
Inotuzumab ozogamicin	CD22 monoclonal antibody-calicheamicin conjugate that binds to CD22-expressing tumor cells and delivers calicheamicin into cells.	CD22	relapsed or refractory B-cell precursor acute lymphoblastic leukemia (ALL)
W6/32	Clone W6/32 recognizes residues in the N terminus of the human ß2-microglobulin molecule	HLA-A/B/C	pancreatic neuroendocrine tumor
87G	87G mAb reacts with isoforms of HLA-G1 and -G5	HLA-G	Melanoma
GHI/75	LILRB1 monoclonal antibody	LILRB1	pancreatic neuroendocrine tumor
HP-F1	LILRB1 monoclonal antibody, full length native protein (purified) corresponding to LILRB1	LILRB1	Triple negative breast cancer
Jd3 scFv	It binds to scFv-conjugated phage (Jd3) with high affinity and against CD24	CD24	Non-small-cell carcinoma
SWA11	SWA11 specifically recognizes the CD24 protein core	CD24	Non-small-cell carcinoma
G7mAb/G7S	G7mAb based on hybridoma technology and then generated a single-chain antibodyfragment (scFv) G7S	CD24	Hepatocellular carcinoma
Ab-2 clone 24C02/SN3b	monoclonal antibody against human CD24	CD24	Gliomas
ALB9	ALB9 targets the LAP sequence present in human, but not the murine homolog CD24	CD24	Breast cancer, Bladder cancer
SN3	high quality monoclonal CD24 antibody (also designated Ly-52 antibody, Nectadrin antibody or M1/69-J11D heat stable antigen antibody	CD24	Breast cancer
HN-01	Anti-CD24-ADC, antibody-nitric oxide conjugate	CD24	Hepatic Carcinoma

Later, the human-murine chimeric antibody ch14.18 was used as a variant of 14.G2a was subsequently renamed dinutuximab. It binds to GD2 and induces ADCC and CDC.^378^ In 2015, the U.S. The Food and Drug Administration (FDA) approved dinutuximab in combination with granulocyte-macrophage colony-stimulating factor, IL-2 and 13-cis retinoic acid (RA) for the treatment of high-grade neuroblastoma in pediatric patients. Dinutuximab is expressed by traditional Sp2/0 cells and contains a Gal-α3Gal glycosylation-modified epitope, which may induce allergy, while the improved version (Ch14.18, named dinutuximab β) expressed in CHO cells had a better glycosylation pattern compared to dinutuximab and avoided virus contamination from mice since it contains almost no Gal-α3Gal; dinutuximab β was approved by the European Medicines Agency (EMA) for high-risk neuroblastoma in 2017.^123^

Humanized mAbs have been developed since chimeric Abs are less immunogenic than murine mAbs. Hu3F8 (Naxitamab) was approved by the FDA for treating neuroblastoma in 2020.^379^ Hu14.18K322A was modified from 14G2a to improve its efficacy and is in phase II clinical trials in children with neuroblastoma.^123^

### CD22 in clinical applications

CD22 undergoes constitutive endocytosis and is well suited for the efficient delivery of toxins into cells.^380^ At present, the drugs targeting CD22 mainly include monoclonal antibody drugs, antibody conjugates (ADC), and CAR-T cells.

Epratuzumab, derived from IgG2 monoclonal antibody (LL2, also called HPB-2), is a humanized IgG1 antibody against CD22 that contributes to BCR signaling by phosphorylating CD22 and induces ADCC.^381^ Epratuzumab has clinical activity and safety, with a 43% objective response rate in follicular NHL patients.^147,381^ SM03, another anti-CD22 recombinant IgG1 mAb, is currently being developed to treat rheumatoid arthritis in a phase III clinical trial.^382^

The FDA has approved two antibody-drug conjugates targeting CD22 to deliver cytotoxic agents to B-cell lymphoma/leukemia cells. Inotuzumab ozogamicin, a calicheamicin-conjugated a monoclonal antibody binding to CD22-expressing tumor cells, can be internalized and release cytotoxic calicheamicin inside the cell, leading to DNA damage and the following cell death.^383^ Inotuzumab ozogamicin was approved for the treatment of adults with refractory or relapsed leukemia. Moxetumomab pasudotox-tdfk, also called HA22 or CAT-8015, an anti-CD22 monoclonal antibody fused to Pseudomonas exotoxin (PE38), is another FDA-approved antibody-drug conjugate for targeting CD22.^384,385^ It is approved for application in patients with refractory or relapsed hairy cell leukemia (HCL). Other ADCs targeting CD22 are in clinical trials. DT2219, a bispecific ligand-directed toxin targeting both CD22 and CD19, is conjugated to the catalytic domain of diphtheria toxin and is used for treating refractory or relapsed B-lineage leukemia or lymphoma.^386^

CD22-targeted and bispecific CARs, such as CD19-CD22 and CD20-CD22 CARs, are the ongoing trials in the treatment of lymphoma and leukemia. Antigen loss is a common cause of resistance to CD19-targeted immunotherapy, but CD22 is also present in most B-ALL cases and is usually retained after CD19 loss.^387^ Therefore, CD22 is a promising candidate for antigen targeting by CAR T cells in patients with CD19 relapse.^388^ CD22 is mostly used as a supplement to CD19 or CD20 CAR T-cell therapy.

In summary, phagocytosis can be realized by either targeting phagocytosis checkpoints or interrupting the binding between ligands and receptors. Furthermore, macrophage activation is modulated by various compounds,^389^ and the compounds library has been built, the related pathway mediating macrophage activation has been elucidated.^389^

## Conclusion and future perspectives

Dual goals of survival improvement and toxicity reduction will be achieved by the promising cancer immunotherapy. Immune checkpoint inhibitors, such as those targeting CTLA-4 and PD-1/PD-L1, have achieved unprecedented clinical applications and ushered in a new phase in the history of cancer treatment. A series of clinical trials have shown that targeting CTLA-4 or PD-1 leads to the proliferation of autoimmune lymphocytes, which increases the risk of adverse autoimmune reactions, such as pneumonia, colitis, hepatitis and vitiligo. Phagocytosis checkpoints have been increasingly recognized, and research on “don’t eat me” signals has also made rapid progress. New phagocytosis checkpoints have been discovered over time, and their evaluations in clinical trials are either in preparation or ongoing.

CD47-SIRPα, as the 1st phagocytosis checkpoint discovered, has already been in clinical trials. Targeting CD47 is less toxic than other approaches; it allows cancer cells to be engulfed by macrophages complelely, with little release of cellular contents after cell death. By enhancing the ADCP of targeted antibodies, disrupting the binding of CD47 to SIRPα has emerged as a promising immunotherapeutic strategy for advanced cancers. Anti-CD47 antibodies are theoretically able to target quiescent tumor stem cells with high expression of CD47.^390^ In addition to CD47, CD24, PD-L1, MHC-I, STC-1 and CD22 are also phagocytosis checkpoints discovered in recent years. Antibodies targeting these phagocytosis checkpoints are in preclinical or clinical trials. From the perspective of targeting phagocytosis, none of these drugs are on the market yet. Some drugs were developed based on other immune responses, but more research will be performed on phagocytosis checkpoint drugs.

Targeting phagocytosis also faces other potential challenges. The function of phagocytosis checkpoints mainly relies on innate responses that are less specific and may induce tissue damage to normal tissues in addition to tumors, especially when phagocytosis targeting is used combing with other immune-modulating methods, such as STING agonists or cytokine therapies. Regarding the CD47 checkpoint, given that CD47 is expressed highly in circulating blood cells, hematotoxicity has emerged as the most common side effect. To mitigate this toxicity, various methods have been developed during the process of antibody and inhibitor development. Presumably, targeting the phagocytosis checkpoint should complement T-cell responses, such as targeting PD-L1, to maximize antitumor responses. Patients who do not respond to anti-PD-L1 therapy may be sensitive to anti-CD47 treatment. More than half of the ongoing clinical trials are combinational therapies targeting CD47, as listed in Tables 3 and 4. In conclusion, targeting phagocytosis checkpoints has ushered in a new era of immunotherapy but also faces new challenges, and further investigation of the mechanisms underlying tumor-mediated immune evasion might overcome these challenges and promote the development of the first drug targeting phagocytosis checkpoints.

**Table 3:** Phagocytosis checkpoint targeting drugs in clinical trial (registered in US) and investigational new drug (IND) stage

Company Name (sponsor)	Drug name	main component	Target	Disease	Clinical phase	Combination drug	single-drug therapy or combination therapy	National Clinical Traial Number (NCT NO.)
Akeso	AK117	Monoclonal Antibody	CD47	MDS	Phase 1/2	Azacitidine	combination therapy	NCT04900350
				AML	Phase 1/2	Azacitidine	combination therapy	NCT04980885
				Advanced Malignancies	Phase 1/2	AK112/Chemotherapy	combination therapy	NCT05214482
				Advanced Malignancies	Phase 1/2	AK112/Carboplatin/Cisplatin/5-Fluorouracil	combination therapy	NCT05229497
				Advanced Malignancies	Phase 1/2	AK104/Capecitabine tablets/Oxaliplatin/ Cisplatin/Paclitaxel/Irinotecan/Docetaxel/5-FU	combination therapy	NCT05235542
				Neoplasms Malignant	Phase 1		single-drug therapy	NCT04728334/ NCT04349969
ALX Oncology	ALX148/evorpacept	Fusion protein	CD47	Gastric Cancer	Phase 2/3	Trastuzumab/Ramucirumab/Paclitaxel	combination therapy	NCT05002127
				NHL	Phase 1/2	Lenalidomide/Biological: Rituximab	combination therapy	NCT05025800
				MDS	Phase 1/2	Azacitidine	combination therapy	NCT04417517
				AML	Phase 1/2	Venetoclax/Drug: Azacitidine	combination therapy	NCT04755244
				Head and Neck Cancer	Phase 2	Pembrolizumab/Cisplatin/Carboplatin; 5FU	combination therapy	NCT04675333
				Head and Neck Cancer	Phase 2	Pembrolizumab	combination therapy	NCT04675294
				MSS Metastatic Colorectal Cancer	Phase 2	Cetuximab/Drug: Pembrolizumab	combination therapy	NCT05167409
				Solid Tumor/NHL	Phase 1	Pembrolizumab/Trastuzumab/Rituximab/ Ramucirumab + Paclitaxel/5-FU + Cisplatin	combination therapy	NCT03013218
Arch Oncology	AO-176	Monoclonal Antibody	CD47	Solid Tumor	Phase 1/2	Paclitaxel/Pembrolizumab	combination therapy	NCT03834948
				Multiple Myeloma	Phase 1/2	Dex/Dex + Bort	combination therapy	NCT04445701
Bio-Thera Solutions	BAT7104	bispecific antibody	CD47/PD-L1	Solid Tumor	Phase 1		single-drug therapy	NCT05200013
Chia Tai Tianqing	TQB2928	Monoclonal Antibody	CD47	Advanced Malignancies	Phase 1		single-drug therapy	NCT05192512
Elpiscience	ES004	Monoclonal antibody	SIRPα	Malignant tumor	IND		/	
EpicentRx	RRx-001	Small molecular	CD47/SIRPα axis	Small Cell Lung Cancer	Phase 3	Cisplatin/carboplatin plus etoposide	combination therapy	NCT03699956
				Colorectal Neoplasms	Phase 2	Regorafenib/Irinotecan	combination therapy	NCT02096354
				Solid Tumor	Phase 2	Cisplatin/Cisplatin/Etoposide/Carboplatin/ Irinotecan/Vinorelbine/Doxil/Gemcitabine/ Taxane/Paclitaxel/ Nab-Paclitaxel/Pemetrexed	combination therapy	NCT02489903
				Oral Mucositis	Phase 2	Cisplatin	combination therapy	NCT03515538
				Cholangiocarcinoma	Phase 2	Gemcitabine and cisplatin	combination therapy	NCT02452970; erminated (Resensitization or clinical benefit was not observed)
				Brain Metastases	Phase 1	WBRT	combination therapy	NCT02215512
				Solid Tumor/Lymphoma	Phase 1		single-drug therapy	NCT02096341
				Progressive Malignant Solid and Central Nervous System Tumors (PIRATE)	Phase 1	Temozolomide/ Irinotecan	combination therapy	NCT04525014
				Metastatic or Advanced Cancer	Phase 1	Irinotecan	combination therapy	NCT02801097
				Solid Tumor/Lymphoma	Phase 1	Nivolumab	combination therapy	NCT02518958
				Glioblastoma and Anaplastic Gliomas	Phase 1	Temozolomide/TMZ	combination therapy	NCT02871843
				Solid Tumor/Lymphoma	Phase 1		single-drug therapy	NCT01359982
Conjupro	CPO107	bispecific antibody	SIRPα/CD20	CD20 Positive NHL	Phase 1/2		single-drug therapy	NCT04853329
GeneScience	Gentulizumab	Monoclonal Antibody	CD47	Solid Tumor/NHL	Phase 1		single-drug therapy	NCT05221385
			CD47	AML/MDS	Phase 1		single-drug therapy	NCT05263271
Gilead Sciences	Magrolimab	Monoclonal Antibody	CD47	HL	Phase 2	Drug: Pembrolizumab/Procedure: PET/CT	combination therapy	NCT04788043
				MDS/AML	Phase 1/2	Drug: Sabatolimab/Drug: Azacitidine	combination therapy	NCT05367401
				Solid Tumor	Phase 1		single-drug therapy	NCT02216409
				Hematological Malignancies	Phase 1	Drug: Azacitidine	combination therapy	NCT03248479
				Lymphoma	Phase 1	Drug: Obinutuzumab/Drug: Venetoclax	combination therapy	NCT04599634
Hengrui Pharmaceuticals	SHR-1603	Monoclonal Antibody	CD47	Nasopharyngeal Carcinoma	Phase1	single and combined withGemcitabine/ Cisplatin/Albumin Paclitaxel	single-drug therapy and combination therapy	NCT04282070
				Solid Tumor	Phase 1		single-drug therapy	NCT03710265
I-MAB	TJC4	monoclonal antibody	CD47	AML/MDS	Phase1	Lemzoparlimab/Azacitidine/Venetoclax	combination therapy	NCT04912063
				Multiple Myeloma	Phase1	Lemzoparlimab/Dexamethasone/ Carfilzomib/Pomalidomide/Daratumumab	combination therapy	NCT04895410
	TJ-011133 (Lemzoparlimab)			Solid Tumor	Phase 1/2	toripalimab	combination therapy	NCT05148533
				AML/MDS	Phase 1/2		single-drug therapy	NCT04202003
				MDS	Phase 1	Azacitidine/Venetoclax	combination therapy	NCT04912063
				Multiple Myeloma	Phase 1	Single or combined with examethasone/ Carfilzomib/Pomalidomide/Daratumumab	single-drug therapy and combination therapy	NCT04895410
				Solid Tumor/Lymphoma	Phase 1	Pembrolizumab/Rituximab	combination therapy	NCT03934814
				MDS	Phase 1	Azacitidine/Venetoclax	combination therapy	NCT04912063
ImmuneOncia	IMC-002	Monoclonal Antibody	CD47	Advanced Malignancies	Phase 1		single-drug therapy	NCT05276310/ NCT04306224
ImmuneOnco Biopharma	IMM-01	Fusion protein	CD47	AML/MDS	Phase 1/2	Azacitidine	combination therapy	NCT05140811
	IMM-2505	bispecific antibody	PD-L1/CD47	Advanced Malignancies	IND		single-drug therapy	IND
	IMM2902	bispecific antibody	CD47/SIRPα	HER2-expressing Advanced Solid Tumor	Phase 1		single-drug therapy	NCT05076591
	IMM0306	bispecific antibody	CD47/CD20	B-NHL	Phase 1		single-drug therapy	NCT04746131
Innovent	SG2501	bispecific antibody	CD38/CD47	Hematological Malignancy	Phase 1		single-drug therapy	NCT05293912
	IBI397	Monoclonal Antibody	SIRPα		IND			IND
	IBI188	Monoclonal Antibody	CD47	Advanced Malignancies	Phase 1		single-drug therapy	NCT03763149/ NCT03717103
	IBI322	bispecific antibody	CD47/PDL1	Advanced Malignancies	Phase 1		single-drug therapy	NCT04338659/ NCT04328831
				Solid Tumor	Phase 1		single-drug therapy	NCT04912466
				Hematological Malignancy	Phase 1		single-drug therapy	NCT04795128
				Myeloid Tumor	Phase 1	HMA	combination therapy	NCT05148442
Haider Mahdi	Pembrolizumab+ ALX148	blocking PD-1 and CD47	CD47/PDL1	Ovarian Cancer	Phase 2	Pembrolizumab+ ALX148	combination therapy	NCT05467670
Baylor College of Medicine	iC9-GD2 T Cells	CAR-T	GD2	Neuroblastoma	Phase 1	iC9-GD2 T Cells/ Cytoxan/Fludara/Keytruda	combination therapy	NCT01822652
University Hospital Southampton NHS Foundation Trust	Ch14.18/CHO	Monoclonal Antibody	GD2	Neuroblastoma	Phase 1	Nivolumab/Ch14.18/CHO		NCT02914405
University of Wisconsin, Madison	hu14.18-IL2	IL-2 linked to hu14.18 mAb	GD2	Melanoma	Phase 2	hu14.18-IL2 combined with Nivolumab/ipilimumab	combination therapy	NCT03958383
JMT BIO	JMT601	Fusion protein	CD20/CD47	NHL	Phase 1/2		single-drug therapy	NCT04853329
				Advanced Malignancies	Phase 1		single-drug therapy	NCT03722186; Suspended (Business Decision)
KAHR Medical	DSP107	bispecific antibody	CD47/41BB	NSCLC	Phase1/2	Atezolizumab	single-drug therapy and combination therapy	NCT04440735
				Hematological Malignancies	Phase1	Azacitidine/Venetoclax	single-drug therapy and combination therapy	NCT04937166
Lunan Pharmacy		Monoclonal antibody	CD47	Malignant tumor	IND		single-drug therapy	/
MABWELL	6MW3211	Bispecific antibody	CD47/PD-L1	Advanced Malignant Neoplasm	Phase1/2		single-drug therapy	NCT05048160
MAB WORKS	MIL-95	Monoclonal antibody	CD47	Advanced Malignancies	Phase 1		single-drug therapy	NCT04651348
OSE Immunotherapeutics	OSE-172	Monoclonal antibody	SIRPα	Solid Tumor	Phase1	BI 754091	single-drug therapy and combination therapy	NCT03990233
				Advanced Cancer	Phase 1	ezabenlimab/[89Zr]Zr- BI 765063	combination therapy	NCT05068102
				HNSCC	Phase1	Ezabenlimab/BI 836880/Cetuximab/ Investigator´s Choice Chemotherapy	combination therapy	NCT05249426
				Solid Tumor	Phase1	BI 754091	single-drug therapy and combination therapy	NCT04653142
Pfizer	PF-07257876	bispecific antibody	CD47/PDL1	Solid Tumor	Phase 1		single-drug therapy	NCT04881045
Seagen	SGN-CD47M	Antibody–Drug Conjugates	CD47	Solid Tumor	Phase 1		single-drug therapy	NCT03957096
Shattuck Labs	SL-172154	bispecific antibody	SIRPα/CD40L	SCC	Phase 1		single-drug therapy	NCT04502888
Sorrento Therapeutics	STI-6643	Monoclonal Antibody	CD47	Solid Tumor	Phase 1		single-drug therapy	NCT04900519
SUNHO (China)	IBC0966	bispecific antibody	CD47/PDL1	Advanced Malignancies	Phase 1/2		single-drug therapy	NCT04980690
Surface Oncology	SRF231^628,629^	Monoclonal Antibody	CD47	Solid Tumor/Hematological Malignancy	Phase1		single-drug therapy	NCT03512340
SUMGEN	SG12473	bispecific antibody	CD47/PD-1	Malignant tumor	Phase 1		single-drug therapy	CTR20211029
	SG2501	bispecific antibody	CD47/CD38	Hematological Malignancy	Phase 1		single-drug therapy	NCT05293912
	SG404	Fusion protein	CD47	Malignant tumor	Phase 1		single-drug therapy	CTR20202489
TG Therapeutics	TG-1801	bispecific antibody	CD47/CD19	Hematological Malignancy	Phase 1	Biological: Ublituximab	combination therapy	NCT04806035
Trillium	TTI-622	Fusion protein	CD47	Solid Tumor	Phase 1/2	Pegylated Liposomal Doxorubicin	combination therapy	NCT05261490
				Leiomyosarcoma	Phase 1/2	Doxorubicin	combination therapy	NCT04996004
				Multiple Myeloma	Phase 1	Daratumumab Hyaluronidase-fihj	combination therapy	NCT05139225
				Hematological Malignancy	Phase 1	Azacitidine/Venetoclax/Carfilzomib/Dexamethasone/ anti-CD20 targeting agent	single-drug therapy and combination therapy	NCT03530683
				Solid Tumor	Phase 1	Monotherapy/Drug: PD-1/PD-L1 Inhibitor/ pegylated interferon-α2a/Other:T-Vec/Other: radiation	combination therapy	NCT02890368
				Advanced Malignancies	Phase 1	Drug: Rituximab/Drug: Nivolumab	combination therapy	NCT02663518
Trillium Therapeutics	TT1-621	Fusion protein	CD47	Leiomyosarcoma	Phase 1/2	Doxorubicin	combination therapy	NCT04996004
				Solid Tumor/Hematological Malignancy	Phase1	Rituximab/Drug: Nivolumab	single-drug therapy and combination therapy	NCT02663518
				Solid Tumor	Phase1	PD-1/PD-L1 Inhibitor/pegylated interferon-α2a /T-Vec/radiation	single-drug therapy and combination therapy	NCT02890368
Waterstone	HX009	bispecific antibody	CD47/PD1	Lymphoma	Phase 2		single-drug therapy	NCT05189093
				Solid Tumor	Phase 1/2		single-drug therapy	NCT04886271/ NCT04097769
ZAI LAB	ZL-1201	Monoclonal Antibody	CD47	Advanced Cancer	Phase 1		single-drug therapy	NCT04257617
Celgene Corporation	Anti-SIRPα	CC-95251	SIRPα	Advanced Solid and Hematologic Cancers	Phase1	Cetuximab, Rituximab	Alone and in Combination with Cetuximab or Rituximab	NCT03783403
Celgene	CC-90002	Monoclonal Antibody	CD47	AML/MDS	Phase1		single-drug therapy	NCT02641002
Insilico Medicine	ISM004-1057D	targeting pyroglutamylation of CD47	CD47 (isoQC)	Solid Tumor/Hematological Malignancy	IND			IND
Nantes University Hospital		Monoclonal Antibody	SIRPα	HCC	Phase 1		single-drug therapy	NCT02868255 completed in Sep.2021
Shandong New Time Pharmaceutical	F527	Monoclonal Antibody	CD47	Lymphoma	Phase 1		single-drug therapy	NCT05293028
SUNHO	IBC0966	bispecific antibody	CD47/PDL1	Advanced Malignant Tumors	Phase 2		single-drug therapy	NCT04980690
Gilead Sciences(Bought Forty Seven in 2020)	Magrolimab (Hu5F9 G4)	Monoclonal Antibody	CD47	Myelodysplastic Syndromes	Phase 3	Azacitidine/Placebo	combination therapy	NCT04313881
				AML	Phase 3	Venetoclax/Azacitidine	combination therapy	NCT05079230
				AML	Phase 3	Azacitidine	combination therapy	NCT04778397
				Hodgkin Lymphoma	Phase 2	Pembrolizumab	combination therapy	NCT04788043
				Solid Tumors	Phase 2	Docetaxel	combination therapy	NCT04827576
				Metastatic Colorectal Cancer	Phase 2	Bevacizumab/Irinotecan/Fluorouracil/Leucovorin	combination therapy	NCT05330429
				Triple-Negative Breast Cancer	Phase 2	Nab-Paclitaxel/Paclitaxel/Sacituzumab govitecan	combination therapy	NCT04958785
				Multiple Myeloma	Phase 2	Daratumumab/Pomalidomide/Dexamethasone/ Bortezomib/Carfilzomib	combination therapy	NCT04892446
				Head and Neck Squamous Cell Carcinoma	Phase 2	pembrolizumab/5-FU/platinum/docetaxel	combination therapy	NCT04854499
				Myeloid Malignancies	Phase 2	venetoclax/azacitidine /mitoxantrone/etoposide/ cytarabine/CC-486	combination therapy	NCT04778410
				Solid Tumor	Phase 1/2	Cetuximab	combination therapy	NCT02953782 finished in Mar.2021
				Non Hodgkin Lymphoma	Phase 1/2	rituximab/gemcitabine/oxaliplatin	combination therapy	NCT02953509
				AML	Phase 1/2	Azacitidine/Venetoclax	combination therapy	NCT04435691
				Urothelial Carcinoma	Phase 1/2	Atezolizumab	combination therapy	NCT03869190
				MDS/AML	Phase 1/2	Sabatolimab/Azacitidine	combination therapy	NCT05367401
				T-Cell Lymphoma	Phase 1/2	mogamulizumab	combination therapy	NCT04541017; Suspended (Other - Amendment Request)
				B-cell Malignancies	Phase 1	Obinutuzumab/Venetoclax	combination therapy	NCT04599634
				Hematological Malignancies	Phase 1	Azacitidine	combination therapy	NCT03248479
				AML	Phase 1	Atezolizumab	combination therapy	NCT03922477
				Ovarian Cancer	Phase 1	Avelumab	combination therapy	NCT03558139 Completed in Dec.2020
				Non-Hodgkin’s Lymphoma	Phase 1	Acalabrutinib/AZD6738/Rituximab/AZD5153	combination therapy	NCT03527147
				Neuroblastoma/Osteosarcoma	Phase 1	Dinutuximab/	combination therapy	NCT04751383
				MDS/AML	Phase 1		single-drug therapy	NCT02678338
				Solid Tumor	Phase 1		single-drug therapy	NCT02216409
				Brain Tumors	Phase 1		single-drug therapy	NCT05169944
Shenzhen Geno-Immune Medical Institute	Sarcoma-specific CAR-T cells	CAR-T	GD2	Sarcoma, Osteoid Sarcoma, Ewing Sarcoma	Phase 2		single-drug therapy	NCT03356782
Xuanwu Hospital, Beijing	GD2-CAR-T cells	CAR-T	GD2	Glioma,Malignant Glioma of Brain,Recurrence Tumor	Phase 1		single-drug therapy	NCT03423992
Biond Biologics	BND-22	Monoclonal Antibody	LILRB1	Advanced Solid Tumors	Phase 1/2	Pembrolizumab	combination therapy	NCT04717375
				Advanced Solid Tumors	Phase 1/2	Cetuximab	combination therapy	NCT04717375
				Advanced Solid Tumors	Phase 1/3		Alone	NCT04717375
NGM Biopharmaceuticals, Inc	NGM707	Dual antibody	LILRB1/LILRB2	Advanced or Metastatic Solid Tumor Malignancies	Phase 1/2		Alone	NCT04913337
					Phase 1/3	pembrolizumab	combination therapy	NCT04913337
Tizona Therapeutics	TTX-080	Monoclonal Antibody	HLA-G	advanced refractory / resistant solid malignancies	Phase 1		Alone	NCT04485013
					Phase 1	pembrolizumab	combination therapy	NCT04485013
					Phase 1	cetuximab	combination therapy	NCT04485013
Janssen Research & Development,	JNJ-78306358	Bispecific antibody binding to CD3 on T cells and human leukocyte antigen G (HLA-G) on cancer cells	HLA-G	Advanced Stage Solid Tumors	Phase 1		single-drug therapy	NCT04991740
Tianhong Li	CD24Fc	CD24 Extracellular Domain-IgG1 Fc Domain Recombinant Fusion Protein CD24Fc	CD24	solid tumors	Phase 1/2		single-drug therapy	NCT04552704(Terminated early by the Sponsor due to the sponsor change.)
OncoImmune, Inc.	CD24Fc	CD24 Extracellular Domain-IgG1 Fc Domain Recombinant Fusion Protein CD24Fc	CD24	Metastatic Melanoma	phase Ib/II	Ipilimumab	combination therapy	NCT04060407 Withdrawn (Business Reasons)
						Nivolumab	combination therapy	
OncoImmune, Inc	CD24Fc	CD24 Extracellular Domain-IgG1 Fc Domain Recombinant Fusion Protein CD24Fc	CD24	Acute Myeloid Leukemia	Phase 3	Methotrexate	combination therapy	NCT04095858 Withdrawn (Business Reasons)
				Acute Lymphoblastic Leukemia		Tacrolimus	combination therapy	
				Myelodysplastic Syndrome			single-drug therapy	
				Hematopoietic Stem Cell Transplantation		Placebo		
				Acute Graft Versus Host Disease				
Tel-Aviv Sourasky Medical Center	EXO-CD24		CD24	SARS-CoV-2	Phase 1		single-drug therapy	NCT04747574
Athens Medical Society	EXO-CD24		CD24	Covid19	Phase 2		single-drug therapy	NCT04902183
Eli Sprecher, MD	Exosomes overexpressing CD24		CD24	COVID-19 Disease	Phase 2		single-drug therapy	NCT04969172
OncoImmune, Inc.	CD24Fc		CD24	Coronavirus Disease 2019 (COVID-19)	Phase 3		single-drug therapy	NCT04317040

**Table 4:** Phagocytosis check point targeting drugs in clinical trial (registered in China) and IND stage

Company Name(sponsor)	Drug name	main component	Target	Disease	Clinical phase	Conbination drug	single-drug therapy or combination therapy	National Clinical Request Number (CTR NO.)
3D Medicines/ImmuneOncia Therapeutics	3D-197/IMC-002	Monoclonal Antibody	CD47	Solid Tumor/Lymphoma	Phase 1		single-drug therapy	CTR20220544
Akeso	AK117	Monoclonal Antibody	CD47	Advanced Malignancies	Phase 1/2	AK112	combination therapy	CTR20220121
				Advanced Malignancies	Phase 1/2	AK112	combination therapy	CTR20212989
				Advanced Malignancies	Phase 1/2	AK104	combination therapy	CTR20220284
BioRay	BR105	Monoclonal Antibody	SIRPα	Advanced Malignancies	Phase 1		single-drug therapy	CTR20220467
Bio-Thera Solutions	BAT7104	bispecific antibody	CD47/PDL1	Advanced Malignancies	Phase 1		single-drug therapy	CTR20220098
Chia Tai Tianqing	TQB2928	Monoclonal Antibody	CD47	Advanced Malignancies	Phase 1		single-drug therapy	CTR20213324
GeneScience	gentulizumab	Monoclonal Antibody	CD47	Hematological Malignancy	Phase 1		single-drug therapy	CTR20210066
Hengrui Pharmaceutical	SHR-1603	Monoclonal Antibody	CD47	Advanced Malignancies	Phase 1		not declared publically	CTR20181964,stopped
ImmuneOnco Biopharma	IMM01	Fusion protein	CD47	Lymphoma	Phase 1		not declared publically	CTR20191531
				HL/B-NHL/AML/MDS/MM	Phase 2		single-drug therapy	CTR20212227
				AML/MDS	Phase 1	Azacitidine	combination therapy	CTR20212519
				Solid Tumor	Phase 1/2	BGB-A317	combination therapy	CTR20220791
	IMM0306	bispecific antibody	CD47/CD20	NHL	Phase 1		not declared publically	CTR20192612
	IMM2902	bispecific antibody	CD47/SIRPα	Solid Tumor	Phase 1		single-drug therapy	CTR20212375
Innovent	IBI188/Letaplimab	Monoclonal Antibody	CD47	Advanced Malignancies	Phase 1		single-drug therapy	CTR20210761
				Advanced Malignancies	Phase 1		not declared publically	CTR20182140
				AML	Phase 1/2	Azacitidine/Decitabine	single-drug therapy and combination therapy	CTR20200938
				MDS	Phase 1/3	Azacitidine	combination therapy	CTR20201039
	IBI322	bispecific antibody	CD47/PDL1	Advanced Malignancies	Phase 1	Bevacizumab/Docetaxel	single-drug therapy and combination therapy	CTR20200175
				Solid Tumor	Phase 1	Sintilimab/Bevacizumab	single-drug therapy and combination therapy	CTR20211251
				Hematological Malignancy	Phase 1		single-drug therapy	CTR20210385
				Myeloid Malignancies	Phase 1		single-drug therapy	CTR20213120
	IBI397			Advanced Malignancies	Phase 1		single-drug therapy	CTR20220193
	ZL-1201	Monoclonal Antibody	CD47	Solid Tumor or Hematological Malignancy	Phase 1		single-drug therapy	CTR20210973
JMT BIO	JMT601	Fusion protein	CD20/CD47	NHL	Phase 1		single-drug therapy	CTR20211365
Mabwell	6MW3211	bispecific antibody	CD47/PDL1	Advanced Malignancies	Phase 1/2		single-drug therapy	CTR20211936
SUMGEN	SG12473	bispecific antibody	CD47/PDL1	Advanced Malignancies	Phase 1		single-drug therapy	CTR20211029
	SG404	Fusion protein	CD47	Advanced Malignancies	Phase 1		single-drug therapy	CTR20202489
SUNHO (China)	IBC0966	bispecific antibody	CD47/PDL1	Advanced Malignancies	Phase 1/2		single-drug therapy	CTR20211609
Waterstone	HX009	bispecific antibody	CD47/PD1	Solid Tumor	Phase 2		single-drug therapy	CTR20211292
				Solid Tumor	Phase 1		not declared publically	CTR20192299
				Lymphoma	Phase 1/2		single-drug therapy	CTR20213391
Shandong New Time Pharmaceutical	F527	Monoclonal Antibody	CD47	Lymphoma	Phase 1		single-drug therapy	CTR20220738
MAB WORKS	MIL95	Monoclonal Antibody	CD47	Solid Tumor or Hematological Malignancy	Phase 1		single-drug therapy	CTR20201108
SUNHO	IBC0966	bispecific antibody	CD47/PDL1	Advanced Malignant Tumors	Phase 2		single-drug therapy	NCT04980690
Shenzhen Geno-Immune Medical Institute	Sarcoma-specific CAR-T cells	CAR-T	GD2	Sarcoma, Osteoid Sarcoma, Ewing Sarcoma	Phase 2		single-drug therapy	NCT03356782
Xuanwu Hospital, Beijing	GD2-CAR-T cells	CAR-T	GD2	Glioma,Malignant Glioma of Brain,Recurrence Tumor	Phase 1		single-drug therapy	NCT03423992

## Data Availability

The datasets of clinical trials in this study are available at the two below websites: The clinical trials registered in the US: https://clinicaltrials.gov. The clinical trials registered in China: http://www.chinadrugtrials.org.cn/m_index.html
